# Integration of breast cancer gene signatures based on graph centrality

**DOI:** 10.1186/1752-0509-5-S3-S10

**Published:** 2011-12-23

**Authors:** Jianxin Wang, Gang Chen, Min Li, Yi Pan

**Affiliations:** 1 School of Information Science and Engineering, Central South University, Changsha, 410083, China; 2 Department of Computer Science, Georgia State University, Atlanta, GA30303, USA

## Abstract

**Background:**

Various gene-expression signatures for breast cancer are available for the prediction of clinical outcome. However due to small overlap between different signatures, it is challenging to integrate existing disjoint signatures to provide a unified insight on the association between gene expression and clinical outcome.

**Results:**

In this paper, we propose a method to integrate different breast cancer gene signatures by using graph centrality in a context-constrained protein interaction network (PIN). The context-constrained PIN for breast cancer is built by integrating complete PIN and various gene signatures reported in literatures. Then, we use graph centralities to quantify the importance of genes to breast cancer. Finally, we get reliable gene signatures that are consisted by the genes with high graph centrality. The genes which are well-known breast cancer genes, such as TP53 and BRCA1, are ranked extremely high in our results. Compared with previous results by functional enrichment analysis, graph centralities, especially the eigenvector centrality and subgraph centrality, based gene signatures are more tightly related to breast cancer. We validate these signatures on genome-wide microarray dataset and found strong association between the expression of these signature genes and pathologic parameters.

**Conclusions:**

In summary, graph centralities provide a novel way to connect different cancer signatures and to understand the mechanism of relationship between gene expression and clinical outcome of breast cancer. Moreover, this method is not only can be used on breast cancer, but also can be used on other gene expression related diseases and drug studies.

## Background

A gene signature is a group of genes whose expression pattern represents the status of a gene expression disease [1]. By using the microarray technology, which has developed rapidly in last ten years, various gene signatures are developed for various complex diseases, especially the cancer. Since researchers found that gene-expression signatures are able to predict clinical outcome of breast cancer in 2002 [2,3], it have become a hot topic and attracted the attention of both biologists and oncologists. Signatures for various phenotypes, such as poor prognosis [3], invasiveness [4], recurrence [5], and metastasis [6,7], have been experimentally derived from patient groups and biological hypotheses. However, distinct signatures share very few genes, even though they paradoxically occupy a common prognosis space. For both cancer biologists and oncologists, a critical problem is whether these disjoint genetic signatures can provide a unified insight on the relationship between gene expression and clinical outcome.

Obviously, complex heterogeneity of signatures caused by different probe design, different platforms, or inadequate patient samples, becomes an obstacle when trying to integrate various signatures of breast cancer. Gene Ontology enrichment, pathway analysis, and some genome-scale methods are proposed to explain the lack of overlap [8-10]. In literature [8], the authors list five possible explanations for the small overlap between signatures:

1. Heterogeneity in expression due to different platform technologies and references;

2. Differences in supervised protocols with which signatures are extracted;

3. Although the genes are not exactly the same, they represent the same set of pathways;

4. Differences in clinical composition between datasets (i.e. sample heterogeneity);

5. Small sample size problems that cause inaccurate signatures.

Through a large-scale analysis that performed on 947 breast cancer samples from Affymetrix platform, the authors of literature [8] conclude that the small signature overlap is most likely due to small sample size problem (explanation 5). However, the conclusion might be specific to the datasets and the specific techniques used in their work. By comparison of three prognostic gene expression signatures for breast cancer, literature [9] suggested that the small overlap between the different prognostic gene signatures is because these different signatures represented largely overlapping biological processes (explanation 3). By taking into account the biological knowledge that exists among different signatures, the authors of [10] found that different signatures are similar at biological level, rather than gene level (explanation 3). Much work has been done in an effort to understand the small overlap between gene signatures, but so far there is no widely accepted explanation.

Meanwhile, computational biologists have developed Protein Interaction Networks(PIN) that effectively have been used to analyze protein interactions underpinning share sub-phenotypes among otherwise seemingly disparate disease, such as retinitis pigmentosa, epithelial ovarian cancer, inflammatory bowel disease, amyotrophic lateral sclerosis, Alzheimer disease, type 2 diabetes, coronary heart disease [11] and head and neck tumor metastasis [12]. For an individual expression signature in breast cancer, protein interaction networks are successfully used to predict prognosis [13] and detect subnetwork signatures of metastatic disease [4]. More recently, in [14], on genome-wide coexpression networks for different disease states, the authors used univariate Cox model and Relief algorithm to select the genes that are the most predictive of clinical outcome to construct gene signature for lung cancer. A 13-gene lung cancer prognosis signature with significant prognostic stratifications is identified by this method. By Single Protein Analysis of Net-works(SPAN [12]) and conservative permutation re-sampling, a small, but more biological significant breast cancer signature consisted by 54 genes is identified from a protein interaction network include 250 cancer-related genes curated from literatures [15,16]. In reference [10], by integrating biological knowledge and different signatures, the authors derived a unified signature that is more robust than original signatures.

However, to integrate different breast cancer signatures, most existing methods need cancer domain knowledge, such as cancer-related literature used in [10,15,16]. This limited the application of these methods. In this paper, we describe a method to integrate different breast cancer signatures by using graph centrality in a context-constrained PIN for human breast cancer which is constructed by integrating disjoint gene signatures reported in previous literatures. Unlike most existing methods, the method proposed in this paper is able to integrate distinct gene signatures without cancer domain knowledge. By Gene Ontology (GO) enrichment analysis, Kyoto Encyclopedia of Genes and Genomes (KEGG) enrichment analysis and relating our results to previous biological studies, we show that the genes in centrality-based signatures are tightly related to breast cancer and are able to predict clinical outcome.

## Methods

**Figure 1:**
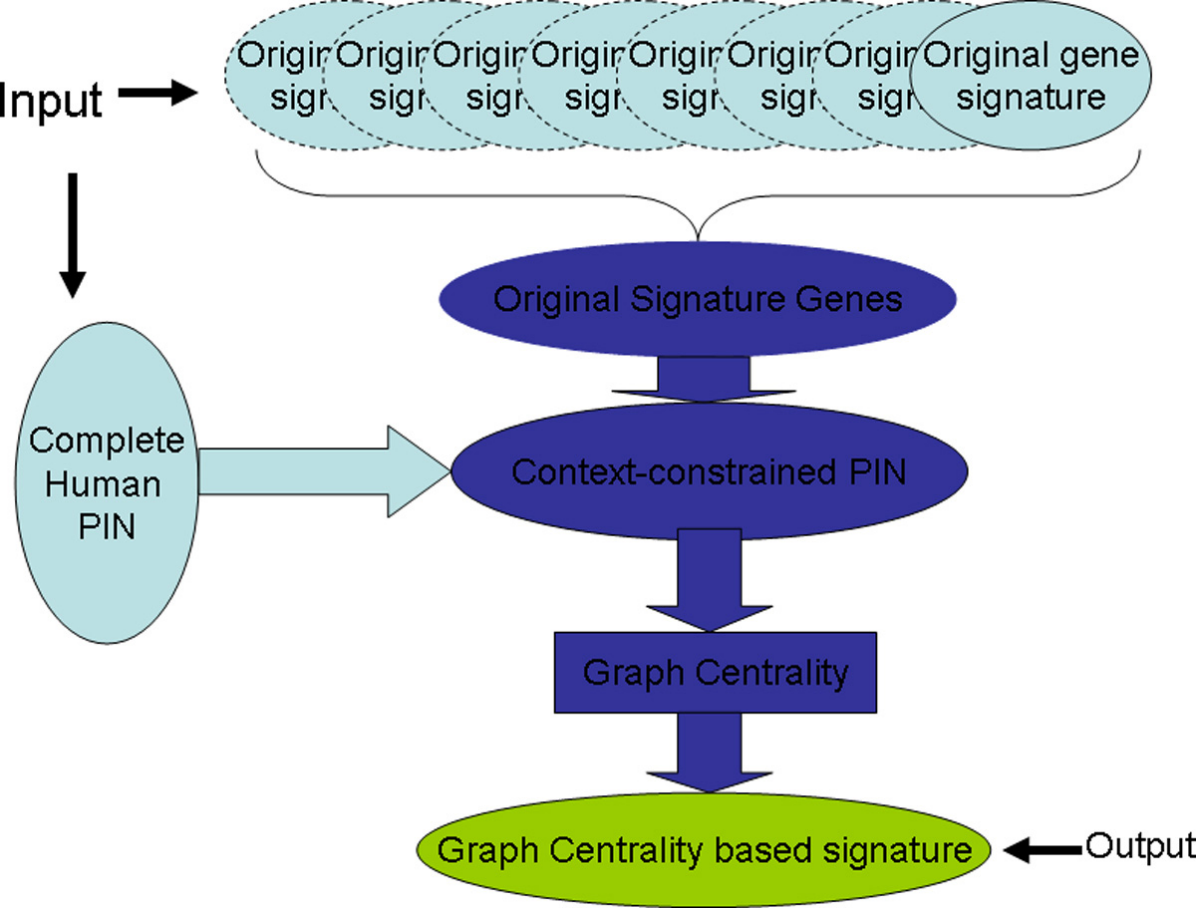
**Schematic overview of graph centrality based integration of gene signatures**. Schematic overview of graph centrality based integration of distinct breast cancer gene signatures.

To identify reliable gene signature of breast cancer by integrating various gene signatures, we propose a graph centrality based method to identify disease genes from a constrained PIN and the overview of this method is provided in Figure 1. Briefly, as shown in Figure 1, the method proposed here has three steps:

1. Collect genes from different breast cancer gene signatures, and discard the genes that exist in only one signature.

2. Project the genes collected in Step 1. to human PIN to construct a context-constrained PIN that consisted. Therefore, to some extent, all genes in this context-constrained network are related to breast cancer. However, we don't know which genes are the most important ones to the breast cancer and can be used to predict clinical outcome.

3. To determine the relationship between genes and breast cancer, we calculated graph centrality of each gene in this constrained PIN. Since the constrained PIN is built based on breast cancer gene signatures, graph centrality of genes in this network indicates their relationship to breast cancer. Output given number of genes with highest graph centrality as the new unified breast cancer signature.

Details of the three steps are described in following three subsections and then validation methods are presented.

### Collecting genes from different signatures

GeneSignDB (http://compbio.dfci.harvard.edu/genesigdb/) [17] is a curated gene signatures database that collected gene signatures for various species and diseases. Keywords "breast cancer" for disease and "human" for species are used to search gene signatures for human breast cancer in GeneSignDB. 94 distinct human breast cancer signatures are obtained, which are reported in 58 different literatures. Since the genes which are included in only one gene signature may be generated by chance, we discard these unreliable genes.

### Construction of context-constrained PIN

A complete human PIN is constructed by integrating protein interaction data from Human Protein Reference Database (HPRD) and BioGRID interaction database [18,19]. After removal of duplicate edges and self-interactions, we got a PIN that is consisted by 51057 distinct interactions among 11465 proteins.

Then, the genes we collected in the first step are projected to the complete human PIN and a constrained PIN for human breast cancer is obtained. This constrained PIN contains 2924 proteins and 4698 interactions.

### Use graph centrality to quantify the relationship between genes and breast cancer

Various definitions of graph centrality have been proposed from different perspectives to evaluate the importance of nodes in a graph. The concept has been widely used in bioinformatics, such as discovery of essential proteins in protein networks [20]. Because it is difficult to infer which definition is best for identifying disease genes in the context-constrained network, we evaluated six different definitions in our work.

For a protein interaction network *G(V,E)*, the six measurements of centrality used in this study are defined as following:

• Degree centrality(*DC*): The degree centrality *DC*(*i*) of vertex *i *is the number of edges connecting node *i *and its neighbors [21].

(1) DC(i)=Deg(i),

where *Deg*(*i*) is the degree of vertexes *i*.

• Betweenness centrality(*BC*): The betweenness centrality *BC*(*i*) of a node *i *is the average fraction of shortest paths that pass through the node *i *[22].

(2) $$BC\left(i\right)=\underset{s}{\sum }\underset{t}{\sum }\frac{\sigma_{st}\left(i\right)}{\sigma_{st}},s\neq t\neq i,$$

where *σ*_*st *_denotes the total number of shortest paths between *s *and *t *and σ_*st*_(*i*) denotes the number of shortest paths from *s *to *t *that pass through the node *i*.

• Closeness centrality(CC): The closeness centrality *CC *of node *i *can defined as [23]:

(3) $$CC\left(i\right)=\frac{1}{\sum_{j\neq i}c_{p}\left(i,j\right)}$$

*CC *is a global metric which describes how the given node *i *connects to other nodes.

• Subgraph centrality(SC): The subgraph centrality *SC*(*i*) of node *i *can be defined as [24]:

(4) $$SC=\overset{\infty }{\underset{l=0}{\sum }}\frac{\mu_{l}\left(i\right)}{l!},$$

where *μ*_*l*_(*i*) denotes the number of closed walks of length *l *which starts and ends at node *i*.

• Eigenvector centrality(EC): The eigenvector centrality *EC*(*i*) of node *i *is defined as the *i*th component of the principal eigenvector of *A*, where *A *is an adjacent matrix. Let λ be an eigenvalue and *e *be the eigenvector. Then for an equation *λe *= *Ae*, we can obtain *EC(i) = e*_1_(*i*), where *e*_1 _corresponds to the largest eigenvalue of *A *[25].

• Information centrality(IC): The information centrality *IC*(*i*) of node *i *in a is defined as [26]:

(5) $$IC\left(i\right)={\left[\frac{1}{n}\underset{j}{\sum }\frac{1}{I_{ij}}\right]}^{-1},$$

where *n *is the number of nodes in graph *G *and *I*_*ij *_= (*r*_*ii *_+ *r*_*jj *_- *r*_*ij*_) - 1, where *r*_*ij *_is the element of matrix *R*. Let *D *be a diagonal matrix of the weighted degree of each node and *J *be a matrix with all its elements equal to one. Then, we get *R *= (*r*_*ij*_) = [*D *- *A*+ *J*] - 1. For computational purposes, *I*_*ii *_is defined as infinite. Thus, $\frac{1}{I_{ii}}=0$.

High centrality of a gene indicates that it is important to the constrained PIN and probably plays an important role in mechanism of breast cancer development. Therefore, according to the graph centrality of genes, we get a gene list that is ordered by the genes' importance to human breast cancer. Depending on specific purpose, a given number of top genes can be selected to construct a reliable gene signature of breast cancer. The reliable gene signature is the integration of the disjoint original signatures.

### KEGG pathway and Gene Ontology enrichment analysis

*p*-value based on the hypergenometirc distribution is widely used as a measurement of the extent to which the clusters are annotated by a specific GO term [27-30]. Basically, the *p*-value is defined as following:

(6) $$P=1-\overset{k-1}{\underset{i=0}{\sum }}\frac{\left(\begin{array}{c} C\\ i \end{array}\right)\left(\begin{array}{ccc} G & - & C\\ n & - & i \end{array}\right)}{\left(\begin{array}{c} G\\ n \end{array}\right)},$$

where *C *is the size of the gene set containing *k *gene with a given GO term; *G *is the size of the universal set of known genes and contains *n *genes with the annotation.

Low *P *in Formula 6 indicates that the module closely corresponds to the GO annotation because the network has a rare chance to produce the module. To simplify our analysis, we define *p*-score as the negative of *log(P) *with the annotation [31].

Gene set enrichment analysis for KEGG pathways is very similar to the one for GO annotations. In Equation 6, *C *is the size of the gene set containing *k *genes that exist in a given KEGG pathway; *G *is the size of the universal set of known genes and contains *n *genes that exist in the pathway. Similarly, *p*-score can be used to measure the relationship between the gene set and a specific KEGG pathway.

In this study, both KEGG and GO enrichment analysis are performed on DAVID [32].

### Validate on microarray dataset

To evaluate the signature's ability to predict clincal outcome, we used expression intensity of the genes in the signature to cluster microarray datasets of breast cancer patients with different pathologic parameters. Patients with similar pathologic parameters should be clustered togather. For a given pathologic parameter, the *p*-value of the clustering result indicates the signature's ability to predict the pathologic parameter.

In this study, euclidean distance between samples are calculated by using the expression intensity of genes in gene signature. Then hierarchical clustering is used to cluster the microarry datasets of breast cancer patients.

## Results

### Overlap among breast cancer signatures

As mentioned in Methods section, 94 breast cancer gene signatures are obtained from public database. Since the different generation methods and the various purposes of these signatures, the size of these signatures are very different. The biggest signature contains 3260 genes, and the smallest signature only contains 4 genes. The median size of these signatures is 46.

To evaluate the similarity among these gene signatures, we analyze the overlap among the 94 gene signatures (see Table 1). The analysis result is very consistent with the results reported in literature [15]. A very small overlap is found among different signatures. 4143 (58.6% of the total number) genes are found in only one signature, but only 24(0.4%) genes overlapped 10 or more signatures, and none of the genes overlapped all 94 signatures. The lack of overlapping is an obstacle to integrate various signatures of breast cancer.

**Table 1:** Overlap among 94 breast cancer gene signatures

Frequency	1	2	3	4	5	6	7	8	9	10	11	12	14	15	16	17
**Num of genes**	4143	1608	687	323	148	56	40	23	15	13	4	3	1	1	1	1

There is very small overlap among distinct breast cancer signatures. Most genes exist in only one signature, and only 24 genes are included in 10 or more signatures.

### Centrality based gene signatures

All genes included in the 94 signatures are projected to the human PIN described in Methods section. In consideration of the lower reliability of gene signatures, only the genes that are included in two or more different signatures are used to construct the context-constrained PIN of breast cancer. Finally, this context-constrained PIN contains 2924 proteins and 4698 interactions.

Then, six graph centralities of each genes in this context-constrained PIN are calculated. Higher centrality for a gene indicates that the gene is more important to this network and should be more tightly related to breast cancer. Graph centrality of each gene in the context-constrained PIN is calculated and provided in Additional File 1. In a recent similar study performed by Chen *et.al*, based on a context-constrained network that obtained from literatures, 10 published gene signatures are integrated and a 54-genes signature is obtained [15]. This result is named as "Chen's signature" in the rest of this paper. For comparison, we also select 54 most important genes identified by each centrality definitions to construct gene signatures. Full list of genes in these graph centrality based signatures are provided in Table 2.

**Table 2:** Graph centrality based breast cancer signatures

BC	CC	DC	EC	IC	SC
TP53	TP53	TP53	TP53	TP53	TP53
EGFR	ESR1	EGFR	ESR1	EGFR	ESR1
CTNNB1	AR	EP300	EP300	EP300	EP300
SMAD3	EGFR	ESR1	AR	ESR1	AR
ESR1	EP300	BRCA1	BRCA1	BRCA1	BRCA1
EP300	SMAD3	CREBBP	CREBBP	CREBBP	CREBBP
SRC	BRCA1	SMAD3	SMAD3	AR	SMAD3
BRCA1	CTNNB1	AR	EGFR	SMAD3	EGFR
CREBBP	SRC	CTNNB1	HDAC1	SRC	HDAC1
AR	CREBBP	SRC	STAT3	CTNNB1	STAT3
UBE2I	AKT1	HDAC1	RB1	HDAC1	RB1
DYNLL1	HDAC1	CASP3	CTNNB1	RB1	CTNNB1
CASP3	STAT3	RB1	JUN	PIK3R1	JUN
ACTB	STAT1	PIK3R1	SRC	CASP3	SRC
AKT1	XRCC6	STAT3	AKT1	STAT3	AKT1
HDAC1	RB1	UBE2I	SMARCA4	AKT1	SMARCA4
PIK3R1	PIK3R1	AKT1	CDKN1A	SHC1	CDKN1A
ZBTB16	PML	CDK2	PML	CDK2	PML
ACVR1	UBE2I	PCNA	STAT1	JUN	STAT1
RB1	HSPA8	SHC1	CDK2	STAT1	CDK2
STAT1	CASP3	JUN	NCOA6	UBE2I	NCOA6
HGS	CDK2	DYNLL1	HDAC2	CDKN1A	HDAC2
CDK2	SMARCA4	STAT1	PIK3R1	PCNA	PIK3R1
YWHAZ	CDKN1A	ACTB	UBE2I	HDAC2	UBE2I
BCL2	JUN	CDKN1A	XRCC6	SMARCA4	XRCC6
XRCC6	CDH1	MAPK14	HIF1A	NFKB1	HIF1A
STAT3	YWHAZ	YWHAZ	NFKB1	CDH1	NFKB1
PLK1	MAPK14	BCL2	CASP3	ACTB	CASP3
PCNA	PRKDC	HDAC2	E2F1	MAPK14	E2F1
HSPA8	HIF1A	ATM	CEBPB	LYN	CEBPB
FN1	SHC1	LYN	PRKDC	XRCC6	PRKDC
VIM	STUB1	NFKB1	SHC1	ATM	SHC1
CD44	HDAC2	XRCC6	NCOA2	ZBTB16	NCOA2
MAPK14	ERBB2	CDH1	CCND1	E2F1	CCND1
GNB2L1	HSPA4	SMARCA4	PCNA	YWHAZ	PCNA
FANCA	BCL2	EZR	TDG	BCL2	TDG
EEF1A1	ZBTB16	HGS	ERBB2	PML	ERBB2
SKIL	LYN	ZBTB16	HSPA4	JAK1	HSPA4
SHC1	NCOA6	PLK1	ZBTB16	EZR	ZBTB16
USP7	PAK1	CAV1	RXRA	PRKDC	RXRA
CAV1	MDM4	E2F1	MAPK14	JAK2	MAPK14
JUN	CASP8	JAK1	JAK2	CASP8	JAK2
RXRA	CEBPB	RXRA	STUB1	RXRA	STUB1
LYN	NFKB1	FN1	CDK7	ERBB2	CDK7
CDKN1A	JAK2	HSPA8	PTPN6	HIF1A	PTPN6
HIF1A	PTPN6	VAV1	ATM	CAV1	ATM
ATM	HGS	ACVR1	BCL3	HSPA8	BCL3
EZR	EZR	CASP8	DDX5	VAV1	DDX5
PPP1CA	CAV1	HIF1A	RBL1	CDKN2A	RBL1
PAK1	PIAS4	JAK2	FOS	NCOA6	FOS
PLSCR1	TDG	PML	CDH1	PTPN6	CDH1
TGFBR2	IGF1R	CDKN2A	MDM4	DYNLL1	MDM4
AURKA	DDX5	EEF1A1	ING1	HGS	ING1
KPNB1	MET	ERBB2	SIN3A	SIN3A	SIN3A

54 genes with highest graph centrality in the context-constrained PIN are selected to consist new breast cancer gene signatures. The genes in the six signatures identified by six graph centrality measurements are listed in this table.

Since all the six graph centrality definitions are designed to measure the importance of a node to a graph, gene signatures identified by the six centrality measurements are similar with each other. A extremely case is all the top 54 genes identified by *EC *and *SC *are the same. By contrast, overlap between our results and Chen's gene signature is only 5-8(9.3%-14.8%, p-value < 0.05).

An interesting result is found in our work. No matter which centrality measurement is used to evaluate the importance of genes in the context-constrained network, TP53 gene, which is already known as a tumor suppressor, is always the most important gene. Another similar example is breast cancer type 1 susceptibility protein (BRCA1). As we expected, our result also shows that BRCA1 plays an important role in the breast cancer. Other similar examples include epidermal growth factor receptor (EGFR), E1A binding protein p300(E300), Androgen receptor gene(AR) and so on (see Table 2).

However, another well-known breast cancer gene, BRCA2, is not included in any signature identified by the six centrality measurement. This is because BRCA2 is included in only one original signature and was discarded when we constructed the context-constrained PIN. On the one hand, the absence of BRCA2 is also a evidence to prove that the quality of existing breast cancer gene signatures is low, and on the other hand, the absence of BRCA2 in out signature indicates that our method can be improved by refining the signature genes collection method.

### Relationship among genes in gene signatures

**Figure 2:**
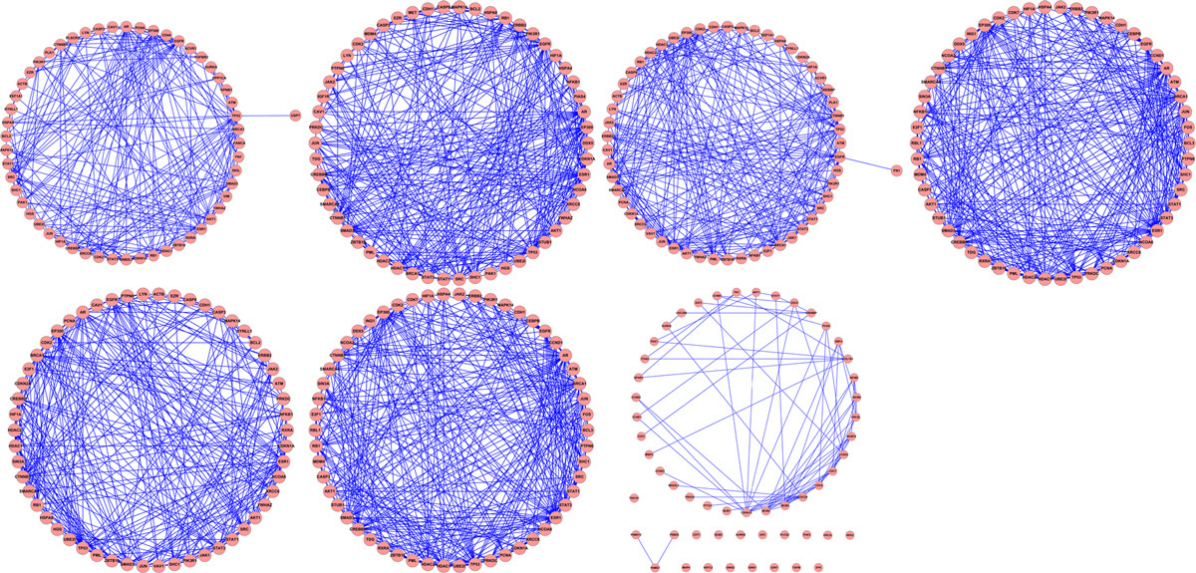
**Sub-networks consisted by the genes of different signatures**. The first six sub-networks are consisted by the signature genes identified by six graph centrality measurements, repectively. The last one is consisted by the signature genes identified by Chen. Compared with Chen's result, all the sub-networks that consisted by the genes of the signatures that identified by graph centrality are connected graph and denser.

To investigate the relationship among genes in the gene signatures, seven sub PINs are constructed by projecting genes in each gene signature to the complete human PIN. As shown in Table 3, the sub PINs consisted by the genes in the graph centrality based gene signatures are much denser than that consisted by the genes in Chen's signature. We also can observe the significant difference in Figure 2. The most dense networks are consisted by the disease genes that are identified by *EC *and *SC*.

**Table 3:** Topological size of context-constrained PINs.

	Proteins	Interactions
BC	54	238
CC	54	330
DC	54	279
EC	54	352
IC	54	312
SC	54	352
Chen	54(35)	70

To investigate the differences between graph centrality based gene signatures and that reported in previous literature, topological size of sub-network consisted by the genes in these gene signatures are calculated and presented in this table. It should be note that the sub-network consisted by the genes included in Chen's gene signature is not a connected graph. The number in parenthesis is the number of proteins of its biggest connected component.

### KEGG pathways enrichment analysis

As shown in Table 4, the most significant KEGG pathway of all six graph centrality-based gene signatures are "pathways in cancer", but that of Chen's gene signature is "Cell cycle". Chen's gene signature is also annotated by "pathways in cancer", but the *p*-score is very low. It is obvious that graph centrality based method is more powerful than SPAN to identify cancer related genes in a constrained PIN.

**Table 4:** KEGG pathways enrichment analysis results.

Method	KEGG Pathway	Description	Annotated Genes	Corrected *p*-score
BC	hsa05200	pathways in cancer	23	13.09
CC	hsa05200	pathways in cancer	29	18.92
DC	hsa05200	pathways in cancer	31	21.59
EC	hsa05200	pathways in cancer	28	25.74
IC	hsa05200	pathways in cancer	30	20.22
SC	hsa05200	pathways in cancer	28	25.74
Chen	hsa04110	Cell cycle	25	26.80
Chen	hsa05200	pathways in cancer	11	2.59

To investigate the relationship between the gene signatures and cancer-related pathways, KEGG pathway enrichment analysis is preformed on these signatures.

### GO enrichment analysis

Biological process GO enrichment analysis is performed on each gene signatures (see Table 5). For each signature, the GO biological process with highest p-score and corresponding p-score are presented in Table 5. Unlike the genes identified in Chen's study [15], which is annotated by term "cell cycle", five centrality-based gene signatures are annotated by "positive regulation of macromolecule metabolic process" and only the gene signature identified by *BC *is annotated by "response to organic substance".

**Table 5:** Gene Ontology enrichment analysis results.

Method	GO Term	Description	*p*-score
BC	GO:0010033	response to organic substance	21.44
CC	GO:0010604	positive regulation of macromolecule metabolic process	21.44
DC	GO:0010604	positive regulation of macromolecule metabolic process	17.50
EC	GO:0010604	positive regulation of macromolecule metabolic process	25.74
IC	GO:0010033	response to organic substance	19.60
SC	GO:0010604	positive regulation of macromolecule metabolic process	25.74
Chen	GO:0007049	cell cycle	19.94

To investigate the relationship between the gene signatures and cancer-related biological processes, Gene Ontology enrichment analysis is preformed on these signatures.

**Figure 3:**
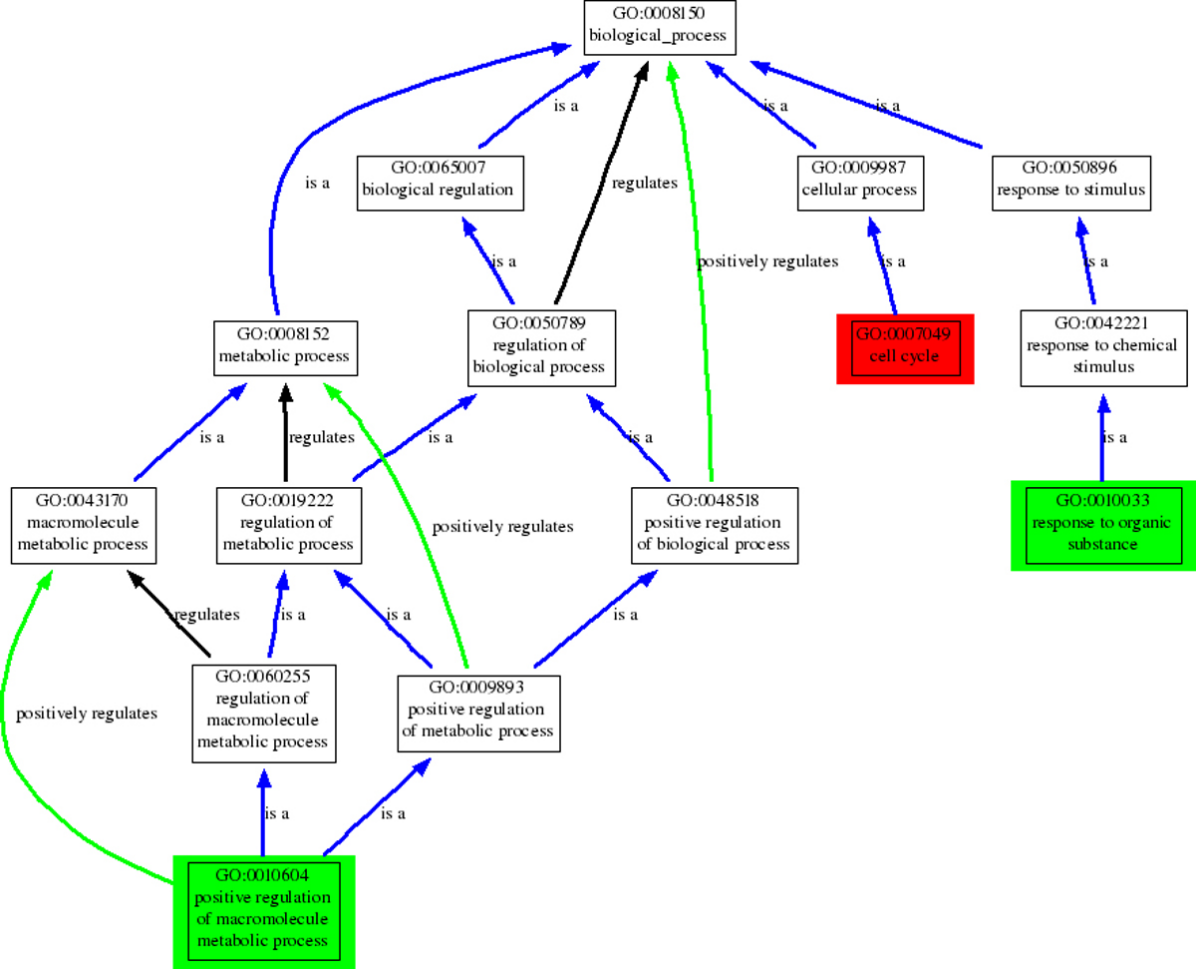
**Relationship among most significant GO terms annotate to significant breast cancer genes identified by different methods**. Relationship among most significant GO terms annotate to significant breast cancer genes identified by different methods. Green boxes indicates the GO terms annotate to genes identified by centrality, and red box indicate the GO term annotate to Chen's results.

The structure of GO terms is a tree-like structure (see Figure 3. The term "regulation of biological regulation", which is located at the same level of "cell cycle", is the ancestor of "positive regulation of macromolecule metabolic process". According to G2SBC database [33], only 190 breast cancer genes are annotated by "Cell Cycle", but 1159 breast cancer genes are annotated by "regulation of biological process". This indicates that the gene signatures identified by graph centrality are probably more tightly related to breast cancer.

According to the *p*-score, both the two types of enrichment analysis suggested that *EC *and *SC *are probably the best choices to identify disease genes from context-constrained network and integrate different gene signatures. As reported in literatures, *SC *also has superior performance in the discovery of essential proteins in protein interaction network [34] and essential proteins tend to have higher correlation with dominant and recessive mutants of disease genes [35]. This is possibly the reason that *SC *and *EC *outperformed other centrality measurements. We also note that the PINs that are consisted by the disease genes identified by *EC *and *SC *are denser than that of other centrality measurements(see Table 3). This indicates that breast cancer genes have tight and complicated relationship with each other.

### Validate of the prognostic potential of the subgraph centrality based gene signature

Since *SC *and *EC *outperform in functional enrichment analysis, we tested the *SC *and *EC *based gene signatures in a genome-wide microarray dataset: GSE7390 [36]. This microarray dataset is downloaded from NCBI GEO database and includes 198 breast cancer patients width different pre-diagnosed pathologic parameters.

We analyze the relationship between the *SC *based signature genes and hormone receptor status using hierarchical clustering. The 198 patients in GSE7390 were divided into two main clusters (Additional File 2), the first cluster includes 118 samples, and the second cluster includes 80 samples. The mean value of each attribute of the samples in each cluster are listed in Table 6. As shown in Table 6, the tumor size and NPI Score of the first cluster is smaller than those of the second cluster. Disease-free survival time, overall survival time, distant metastasis-free survival time, 10-year overall survival probability and time to distant metastasis of the patients in the first cluster is longer than those of the patients in the second cluster. In another word, the condition of patients in the first cluster is much better than the second cluster of patients.

**Table 6:** Clinical outcome of two main clusters

	118 samples cluster	80 samples cluster
Patient Age	47	45
Tumor Size(mm)	2.06	2.36
Disease-free survival (days)	3498	3252
Overall survival (days)	4391	3792
Distant metastasis-free survival (days)	4148	3667
Time to distant metastasis (days)	4148	3667
NPI Score	3.4	4.17
10-year overall survival probability	83.38	74.98

Mean value of each clinical outcome recorded in the microarray dataset of the samples in each cluster is presented in this table.

In the first cluster of patients, 104 of them were ER+; and in the second cluster of patients, only 30 of them were ER+. The *p*-value calculated by ANOVA is 2.47 × 10^-13^. We also explored the relationship between the signature genes and the time to distant metastasis. In the first cluster, the time to distant metastasis of 97 patients were longer than 2000 days; and in the second cluster, that number is only 55. The *p*-value is 0.04254. Normally, *p*-value that smaller than 0.05 means the result is statistic significant and is not generated by chance. Such small *p*-value of the clustering result indicates that the *SC *based signature is able to predict the clinical outcome very well.

## Discussion and conclusion

Identification of genes which play important roles in the development of cancer is a critical problem needed to solve in current research of various cancers. Gene expression signatures provide a way to find significant cancer genes in given groups of patients. Due to the low overlap between heterogeneous signatures, how to integrate them is becoming a serious problem. Fortunately, as shown in this paper, graph centralities, especially *EC *and *SC*, are useful tools to integrate existing different cancer gene signatures.

As well-known, weighted protein interaction network can be constructed by integrating functional annotations, and centrality is also can be extended to weighted network easily [20]. More promising results should be found in the weighted protein interaction network. Besides this, other topological parameters from graph theory may improve this method as well.

## Competing interests

The authors declare that they have no competing interests.

## Authors' contributions

GC conceived and carried out this work under the guidance and supervision of JW. JW, GC and ML drafted the manuscript together. YP participated in revising the draft. All authors have read and approved the manuscript.

## Supplementary Material

Additional file 1 **Graph centrality of genes in the context-constrained PIN**. In this study, graph centrality of each gene in the context-constrained PIN is calculated and used to quantify the relationship between genes and the breast cancer. The calculation results are provided in this additional file. Click here for file

Additional file 2 **SC-based gene signature based hierarchical clustering analysis of breast cancer microarray dataset by using SC-based gene signature**. According to the gene signature identified by SC, hierarchical clustering analysis is performed on the breast cancer microarray dataset, GSE7390, which include 198 breast cancer patients with various pathologic parameters. Click here for file
