# Supplementary material for: Integration of breast cancer gene signatures based on graph centrality
Source: BMC Syst Biol. 2011 Dec 23;5(Suppl 3):S10. doi: 10.1186/1752-0509-5-S3-S10 (PMC3287565; doi:10.1186/1752-0509-5-S3-S10)
Supplement: Additional file 1 — Graph centrality of genes in the context-constrained PIN. In this study, graph centrality of each gene in the context-constrained PIN is calculated and used to quantify the relationship between genes and the breast cancer. The calculation results are provided in this additional file. [file 1752-0509-5-S3-S10-S1.pdf]

Protein	DC	IC	EC	SC	BC	CC
A2M	10	1.25126206	0.000742736	2649.087392	11715.50445	0.020351957
AATF	8	1.26092736	0.016720741	1300794.312	2041.679481	0.020585859
ABCD3	4	0.83856383	7.69928E-05	32.96310861	4154.75561	0.020010785
ABCF1	1	0.56781426	0.000216715	221.8452422	0	0.020137791
ABL2	1	0.59045418	0.002442529	27985.29818	0	0.020319252
ABLIM1	3	0.97981683	0.001794698	14989.40251	1672.415643	0.020415241
ACACA	2	0.81545661	0.009425703	413176.7084	527.3420689	0.020505243
ACIN1	1	0.59740928	0.003989151	74000.41531	0	0.020423302
ACTA2	4	1.07785792	0.019059038	1688863.522	684.2070559	0.020574445
ACTB	39	1.45085544	0.031514982	4617024.471	90036.41528	0.020771224
ACTG1	15	1.34855334	0.009457009	416282.6594	26341.1536	0.020644116
ACTL6A	9	1.28801039	0.015851375	1168312.377	1115.702037	0.020535091
ACTN1	12	1.32286992	0.016657876	1294437.901	14986.76618	0.020621919
ACVR1	26	1.32695832	0.018725195	1629852.16	70987.11366	0.020663108
ADAM11	1	0.43743604	1.27813E-05	2.420849089	0	0.019778463
ADAM12	5	1.13860301	0.011207794	589331.2145	2539.838682	0.020575437
ADAM15	8	1.24572019	0.00997906	466833.2471	6580.937612	0.02057147
ADAM17	5	1.11677764	0.002522057	29647.95737	2566.482793	0.020392316
ADAM9	4	1.02394321	0.000226232	253.1188312	593.9809252	0.020133278
ADAMTS1	3	0.96227632	0.000462065	1005.649358	773.0803826	0.020260647
ADCY6	1	0.47382686	5.0275E-05	13.54641143	0	0.019899048
ADD1	2	0.83351741	0.004493168	93866.05725	147.8359244	0.020436016
ADM	2	0.71131031	0.00055746	1489.718925	0	0.020164672
ADORA2B	1	0.59043667	0.001611439	12255.54147	0	0.02036847
ADRA2A	2	0.81494353	0.002819793	36968.62007	59.76086231	0.02042599
ADRM1	8	1.18177623	0.008004436	299841.8305	7288.588275	0.020567256
AGFG1	4	0.5438089	0.000523168	1292.268137	9960	0.020222962
AGR3	1	0.49139813	5.38277E-05	16.06722859	0	0.01988097
AGT	3	0.51440043	0.000576506	1564.930195	6642	0.020211708
AGTR1	5	1.05462747	0.012780943	763126.2185	12263.37568	0.020616191
AHCYL1	3	0.94328922	0.002057016	19754.21756	3461.792307	0.020412312
AHNAK	4	1.09951529	0.015408519	1106577.084	816.6381708	0.020656857
AHSA1	2	0.75302784	0.006836901	219234.2271	799.2324305	0.020534597
AIP	5	1.05903141	0.010086577	474701.2123	4164.164229	0.020584866
AK5	1	1.11023275	-8.03282E-17	1.543080635	0	0.000585823
AKAP11	1	0.58538039	0.002247375	23509.72524	0	0.020353655
AKAP8	5	1.15578794	0.01252193	728978.3825	424.7237785	0.020546214
AKR7A2	1	1.11023275	8.63704E-33	1.543080635	0	0.000585823
AKR7A3	1	1.11023275	-1.10977E-33	1.543080635	0	0.000585823
AKT1	46	1.47549069	0.111992117	58296086.41	86977.62785	0.020882011
AKT2	5	1.12659404	0.019794155	1820992.564	332.4555248	0.020614946
ALCAM	1	0.60093811	0.006706974	211028.0891	0	0.020533609
ALDOA	4	1.09414508	0.010788099	542971.7064	4269.449794	0.020630892
AMBRA1	3	0.90900002	0.002069808	19930.70984	102.4027648	0.020297265
AMFR	5	0.99209859	0.000723453	2446.04493	3660.35722	0.020281831
ANG	2	0.35422278	1.40925E-05	3.176002285	3322	0.019768156
ANKRD28	1	0.58271549	0.001291238	7761.157645	0	0.020323364
ANKRD44	1	0.58271549	0.001291238	7761.157645	0	0.020323364

ANXA1	2	0.86247573	0.012803101	767715.4344	0	0.020582384
ANXA5	4	1.06654166	0.000854019	3418.33932	2475.09874	0.020369199
ANXA7	1	0.43369928	2.58691E-05	4.778199731	0	0.019836151
ANXA9	1	0.45124314	4.29704E-05	10.42810934	0	0.019947415
AP2A2	5	1.13532998	0.007505491	262624.5827	3283.556856	0.020542999
AP2B1	9	1.22814985	0.006571504	200823.5253	16081.97875	0.020552151
AP2S1	2	0.55210687	0.00029582	410.3543719	3322	0.020149677
APBB3	1	0.54346554	0.00020261	193.9163662	0	0.019994143
APC2	5	1.14129508	0.011438111	608665.9889	754.015289	0.020553141
APH1B	1	0.57769367	0.001291789	7798.523312	0	0.02030644
APLP1	8	1.18788761	0.004509989	94768.16613	4223.719699	0.020390855
APOD	2	0.81192359	0.001062549	5270.264128	62.16154782	0.020262811
APOE	8	1.21081159	0.001345163	8446.823953	7165.979769	0.020386715
AQP1	2	0.82004358	0.000553975	1439.764061	215.0895621	0.020217214
AQP3	1	0.58968641	0.00253384	30102.48911	0	0.020366526
AR	63	1.49205693	0.212126131	209108786.4	106452.379	0.020960731
AREG	3	0.99168669	0.008147009	310367.7179	3462.182142	0.020584866
ARF1	5	1.08752971	0.003704625	63795.12945	1651.891092	0.020471062
ARFGEF1	1	1.11023275	6.26317E-33	1.543080635	0	0.000585823
ARFGEF2	1	1.11023275	-3.38017E-34	1.543080635	0	0.000585823
ARHGAP5	2	0.76098203	0.000636776	1934.687224	109.5563144	0.020210751
ARHGDIA	16	1.36658196	0.013957126	909861.5337	7996.340445	0.020605241
ARHGDIB	4	1.0811165	0.008084189	304908.0872	453.6467323	0.020542999
ARHGEF7	4	1.07698991	0.004497937	94792.41852	292.7447444	0.020483345
ARID1A	4	1.08196005	0.011379157	602288.2306	6.498385892	0.020467871
ARL3	2	0.80605848	0.011548589	620704.9822	443.7276107	0.02058288
ARL6IP1	1	0.58823865	0.001673396	13089.82011	0	0.020370658
ARL6IP5	1	1.11023275	4.00794E-17	1.543080635	0	0.000585823
ARMCX2	1	0.58690834	0.001236071	7108.870307	0	0.020330626
ARNT	9	1.29896146	0.049124811	11214981.38	2242.625281	0.020715517
ARPC3	3	0.97175081	0.003208149	47838.02852	483.9608259	0.020444583
ARPC5	2	0.76831064	0.000805207	3027.934952	161.5884897	0.020222722
ASB8	2	0.76751859	0.000360949	611.9069738	0	0.020083771
ASF1B	2	0.78721083	0.001416652	9343.357663	109.659917	0.02036847
ASH2L	3	0.98589793	0.009225225	395724.0107	24.93830382	0.02052324
ASPM	2	0.85475252	0.012436736	720014.8818	0	0.020578166
ATAD2	1	0.60017776	0.009919944	457388.8922	0	0.020548935
ATF3	7	1.23699785	0.030646776	4367006.232	3833.275703	0.02068013
ATF5	3	0.89283677	0.00168589	13223.57433	407.1374684	0.020258483
ATF7IP	14	1.32089378	0.00371886	64544.6909	26077.01997	0.020549924
ATM	33	1.44502667	0.061144549	17409666.04	37651.7708	0.02071426
ATP2A2	4	1.06645033	0.005713397	152126.4248	3144.477275	0.020545719
ATP5A1	1	0.59087769	0.00238292	26408.51754	0	0.020401578
ATP5B	2	0.82635389	0.002505669	29223.51133	246.0947262	0.02038209
ATP6AP1	1	0.26169141	6.331E-07	1.592784646	0	0.019395082
ATP6V0C	1	1.11023275	-4.25972E-17	1.543080635	0	0.000585823
ATRIP	4	1.06745541	0.004834315	109141.7263	91.40518776	0.020344195
AURKA	20	1.37366105	0.035254401	5783122.131	36040.2016	0.020683388
AURKB	8	1.15232109	0.00312209	45418.11947	4412.780186	0.020365068

AXL	7	1.16165842	0.014885027	1042228.706	6229.04842	0.020553636
AZI2	2	0.64828826	0.000144998	100.6952403	90.24525574	0.020011723
AZIN1	1	0.58241143	0.0014783	10170.02802	0	0.020282554
Actb	2	0.80534876	0.001060893	5252.502503	454.3290276	0.020345407
Actg1	1	0.58829399	0.000887419	3676.462767	0	0.020317317
Actl6a	1	0.58258102	0.002498382	29045.19093	0	0.020280626
Akt2	1	0.59667537	0.005031206	117691.5856	0	0.020466154
Aldoa	1	0.58829399	0.000887419	3676.462767	0	0.020317317
Ap2b1	1	0.57083629	0.000841223	3295.020352	0	0.020255838
Apc2	1	0.59873321	0.006392688	190190.6433	0	0.020493181
Ar	1	0.59873321	0.006392688	190190.6433	0	0.020493181
Arhgef7	1	0.59914918	0.00745681	258503.9824	0	0.020517813
B2M	6	1.05141967	0.001216058	6915.840977	5482.199104	0.02035851
BAG1	14	1.37261015	0.056683884	14931554.42	6569.373569	0.020755818
BAG4	3	0.99886646	0.00783101	285051.6231	176.8838465	0.020526449
BARD1	13	1.35555369	0.038752223	6987106.076	7774.191037	0.020694923
BAT3	13	1.29098535	0.013146141	803215.4625	28867.18254	0.020647612
BAX	7	1.17278137	0.005444881	137990.8037	825.8921551	0.020464681
BBC3	4	1.02651719	0.002921825	39746.49541	213.3722115	0.020402309
BCAM	2	0.76847723	0.001088392	5516.238042	630.1256533	0.020328689
BCAP31	8	1.22840627	0.009432938	413973.9446	11827.58054	0.020582632
BCAS1	1	0.58829399	0.000887419	3676.462767	0	0.020317317
BCKDK	1	0.55314558	0.000435613	884.3488405	0	0.020190431
BCL10	9	1.21224007	0.008726035	354345.9234	8850.10851	0.020552893
BCL11A	1	0.4633735	0.00073074	2487.054211	0	0.020160385
BCL2	34	1.43921477	0.048735901	11041041.71	65190.82399	0.020802359
BCL2A1	5	1.06070723	0.001189934	6610.166758	40.00434834	0.020237107
BCL2L11	5	1.13438372	0.008595841	343495.0231	2658.372015	0.020585362
BCL3	15	1.37448645	0.061013179	17300587.87	8727.182953	0.020732626
BECN1	2	0.7824884	0.002282429	24236.84802	319.0325142	0.020394996
BEX2	1	0.5104302	0.000246573	285.4081961	0	0.020093937
BGN	5	1.10232683	0.000689526	2379.868427	1647.672148	0.020224639
BHLHE40	2	0.85445554	0.007732414	278048.1131	292.962205	0.020533609
BICD2	2	0.58353289	0.001293855	7793.532452	6640	0.020324332
BIN1	6	1.10106807	0.004736345	104276.5707	6922.269348	0.020512143
BIRC3	4	1.04362599	0.005064702	119269.6471	2036.830488	0.020483099
BIRC5	5	1.07849894	0.008756628	356556.8862	5537.410854	0.02052176
BLM	20	1.40369021	0.049241191	11288709.57	8339.51673	0.020693167
BMI1	4	1.05728166	0.007935776	292841.3415	1130.573469	0.020507953
BMP1	4	0.95784244	0.000408868	843.0041828	3435.507157	0.020175874
BNIP3L	1	0.59065493	0.002189443	22302.08792	0	0.020389637
BRAP	3	0.95916427	0.010231835	486906.0907	5246.544621	0.020531386
BRCA1	69	1.49541725	0.207767958	200670889.7	117980.0072	0.020915785
BRF2	9	1.14515876	0.000957012	4281.466993	12327.32374	0.020325784
BSG	5	1.04751371	0.004350035	88651.36839	5708.208428	0.020474745
BTF3	1	0.53792511	0.001482165	10215.53846	0	0.020244067
BTG2	4	0.93002216	0.0026715	33181.73922	3516.14328	0.020365797
BTG3	1	0.52918257	0.000432245	872.8071729	0	0.020090389
BUB1	5	1.14832114	0.009557215	425047.5395	4332.050319	0.020555121

BUB1B	8	1.22988164	0.010500851	513095.7686	3257.777613	0.020541269
BYSL	2	0.56560002	0.000605158	1708.074262	9954	0.020171821
Bat3	1	0.47867268	8.4057E-05	35.07901903	0	0.020002812
Bcl2l11	4	1.05625299	0.003564635	59127.90751	605.1349598	0.020470817
Becn1	1	0.59065493	0.002189443	22302.08792	0	0.020389637
Bmi1	2	0.80997879	0.003553383	58698.08246	162.250531	0.020406455
Bub1	1	0.56543911	0.000248321	289.9871905	0	0.020137554
C10orf10	3	0.94926423	0.001311409	8059.156284	355.4419334	0.020343467
C10orf119	2	0.83112801	0.001821877	15439.73724	176.9023844	0.020342255
C11orf58	1	0.55635743	3.33672E-05	7.895356805	0	0.019956743
C12orf10	1	0.58282536	0.001231304	7053.65598	0	0.020302093
C15orf23	1	0.53688957	0.000763449	2719.770769	0	0.020206205
C17orf28	2	0.77318952	0.00206877	19928.06088	165.188037	0.020349773
C1QBP	3	0.92327055	0.00073794	2536.778529	5526.583025	0.020267142
C1R	1	1.11023275	-5.10968E-33	1.543080635	0	0.000585823
C1S	1	1.11023275	5.34681E-33	1.543080635	0	0.000585823
C1orf174	1	0.44687465	0.00011665	65.25714786	0	0.019985014
C20orf20	8	1.24357107	0.008165152	309970.3755	2231.146666	0.020552646
C20orf24	4	0.8575945	0.000168436	137.4011339	4274.768293	0.020067243
C4A	1	0.48664429	1.07024E-05	2.4208245	0	0.019739353
C6orf211	1	0.54267375	0.000507484	1204.234047	0	0.020088261
C9orf89	1	0.54850673	0.000392014	718.5936033	0	0.020149914
CA9	3	0.98919161	0.009764577	444106.6427	0	0.020530646
CABC1	1	0.50276592	6.75713E-05	23.15492074	0	0.01997753
CABLES2	1	0.60238036	0.011145498	578362.3686	0	0.020555863
CAD	2	0.85346933	0.006236526	180835.7668	81.20502294	0.02047843
CADM1	1	0.56971106	0.000167068	133.8829339	0	0.020147061
CALCOCO1	4	1.08113337	0.018651837	1617477.166	2743.400028	0.020571966
CALD1	1	0.59636493	0.003730592	65960.5962	0	0.020398896
CALML5	1	0.53761669	0.000173121	142.3555023	0	0.02006064
CAMLG	1	0.60093811	0.006706974	211028.0891	0	0.020533609
CANX	7	1.15350023	0.002188239	22316.1921	4674.381126	0.020379899
CAP1	5	0.91667627	0.001871067	16317.3069	6941.165209	0.020399871
CAP2	2	0.76292373	0.00050891	1215.333942	0	0.020238307
CAPN2	1	0.59065493	0.002189443	22302.08792	0	0.020389637
CAPZA1	2	0.79079324	0.0014551	9860.029281	899.24685	0.020363853
CARD10	1	0.54850673	0.000392014	718.5936033	0	0.020149914
CASC3	2	0.70354383	0.000318231	473.8949141	186.7796981	0.020121175
CASP10	10	1.30744385	0.017789999	1472807.405	1850.443149	0.020625906
CASP3	55	1.47998668	0.088796496	36646978.2	95340.28025	0.020837402
CASP8	26	1.43630033	0.047008218	10276909.05	29018.2113	0.020787666
CASP8AP2	3	0.98767206	0.00396877	73572.03139	0	0.020458304
CAV1	28	1.43347798	0.056402001	14845902.62	43648.37573	0.020778304
CBFA2T2	6	1.16368696	0.006923143	223130.3083	1797.467084	0.020433081
CBFA2T3	6	1.16910344	0.014755985	1013240.494	1120.547789	0.020509925
CBS	3	0.90387283	0.001924137	17273.93285	1165.640982	0.020403041
CBX7	1	0.52184779	0.000490942	1125.171972	0	0.020105297
CCL13	2	0.80204848	0.000161683	132.4817002	12.422447	0.02003286
CCL18	2	0.853649	0.011675103	634560.0908	1073.163718	0.02060176

CCL2	6	1.13386756	0.000894963	3783.514749	1533.738035	0.020234948
CCL20	1	0.55681109	7.62818E-05	30.89473082	0	0.019958843
CCL5	8	1.18652972	0.000895325	3778.591906	2143.998423	0.020237827
CCL8	3	0.94667129	0.000743111	2595.192227	163.0938976	0.020215059
CCNA2	20	1.41225638	0.055430112	14301035.56	6431.965406	0.020696428
CCNB1	18	1.39213872	0.037369189	6493789.048	12214.52107	0.020707475
CCNB2	4	1.09420558	0.011560585	621714.4045	566.7077361	0.020572957
CCNC	4	1.07084794	0.019402969	1749657.267	1091.69008	0.020619927
CCND1	21	1.41381557	0.081372973	30775865.2	13042.7595	0.020764402
CCND3	14	1.3510361	0.028927551	3891330.81	10162.9189	0.020633635
CCNE1	17	1.39493312	0.053211718	13168781.29	4557.615512	0.020681884
CCNE2	2	0.85712688	0.009234186	396789.8684	0	0.020494411
CCNF	2	0.84280564	0.003909984	71164.08642	0	0.020414753
CCNG2	1	0.37557337	0.000432522	871.9389341	0	0.020119515
CCNO	1	0.59469486	0.003589563	60056.60715	0	0.020357539
CCR1	6	1.17254125	0.013646717	867401.9851	8775.46926	0.020537068
CCT4	8	1.2321148	0.006560157	200191.5178	1606.648205	0.020513375
CCT5	8	1.23414987	0.015315137	1091169.874	4895.864467	0.020647612
CCT6A	5	1.0923947	0.001897619	16854.32557	0	0.020331594
CCT7	5	1.0923947	0.001897619	16854.32557	0	0.020331594
CD2	6	1.07166673	0.001281399	7819.724189	4864.595505	0.020288822
CD2BP2	11	1.25278336	0.002407908	27001.94916	9729.092369	0.020419393
CD34	2	0.77295409	0.001113916	5911.786991	284.7682025	0.020261849
CD38	1	0.54350408	0.000139154	94.6338955	0	0.02003333
CD44	24	1.41623235	0.027629416	3583333.66	51413.325	0.020719791
CD47	3	0.98072373	0.00129696	7958.95582	837.3366369	0.020374305
CD48	1	0.51777563	5.75664E-05	18.07364087	0	0.019896032
CD53	2	0.77538474	0.00019672	189.1420771	89.07961566	0.020070547
CD59	4	0.95374706	0.006305573	186033.3402	5499.921032	0.020499826
CD74	4	0.96121198	0.002023958	19161.47602	4425.971887	0.020432102
CD79A	6	1.13742563	0.007834403	288034.3195	4388.421504	0.02051658
CD81	6	1.12574359	0.008226179	316677.7925	6838.066834	0.020525708
CD9	9	1.18807173	0.003097493	45181.38362	7909.059427	0.020431613
CD99	2	0.82160885	0.001564134	11381.82002	612.7035323	0.02036604
CDC16	9	1.2713967	0.015309096	1089364.02	2121.301863	0.020632139
CDC2	20	1.40257198	0.049665084	11470284.61	21331.32701	0.020758847
CDC20	14	1.34976666	0.024432855	2777988.11	8371.202619	0.02060549
CDC25A	10	1.31671879	0.040357081	7569077.811	3065.639233	0.020744972
CDC25B	11	1.33302594	0.044971058	9398595.296	5971.900895	0.020721552
CDC25C	10	1.32154017	0.036158046	6077075.485	5503.31573	0.020748754
CDC27	14	1.35848627	0.032232928	4829455.883	6527.835175	0.02070647
CDC2L2	5	1.15662569	0.009413579	412150.6732	2406.378338	0.020591074
CDC37	7	1.24104424	0.027042651	3398680.23	8393.696574	0.020671866
CDC42BPA	2	0.45584408	0.000516473	1246.340568	3322	0.020170629
CDC42EP4	3	0.36237951	1.12464E-05	3.644860923	6642	0.019753286
CDC45L	7	1.22418099	0.009546921	424953.2249	231.6722648	0.020474254
CDC6	16	1.37595142	0.025268518	2976323.26	6956.306973	0.020605987
CDC7	12	1.3116724	0.015906825	1179593.463	4196.638621	0.020548193
CDCA3	3	0.83092313	0.001829177	15572.36441	3322	0.02030934

CDCA5	1	0.59213255	0.004147801	79987.71409	0	0.020445807
CDCA8	2	0.76143063	0.000533647	1330.157848	0	0.020139929
CDH1	32	1.45092345	0.060135018	16831879.97	34648.70835	0.020822152
CDH3	4	1.08238041	0.011308941	596122.8181	0	0.020534103
CDK2	46	1.4728606	0.101765169	48160104.54	67618.75561	0.020837148
CDK2AP1	2	0.70459099	0.004582102	97735.7313	2691.199008	0.020426479
CDK2AP2	2	0.59749704	0.000230106	249.3213806	105.2980522	0.020083534
CDK5RAP3	3	0.60041194	0.008502331	336129.5564	6642	0.020473272
CDK6	16	1.37886218	0.048652259	11004426.68	9206.773282	0.020703457
CDK7	16	1.38113742	0.063536242	18768427.73	7319.768661	0.02075708
CDKN1A	37	1.46437775	0.103783159	50075868.27	40564.43035	0.020833333
CDKN2A	25	1.42871891	0.04583007	9776746.299	9447.244496	0.020691912
CDKN2C	5	1.12700769	0.015693396	1146688.933	1620.776301	0.020588838
CDKN2D	1	0.58023211	0.002185686	22226.79679	0	0.020294611
CDKN3	3	0.99160837	0.008615978	345322.0899	18.32132323	0.020499826
CEBPB	21	1.41904167	0.086021811	34389758.17	11463.50107	0.020787413
CEBPG	5	1.04879355	0.007485594	260515.8089	3851.247367	0.020414509
CELSR1	1	0.37538483	0.00042017	823.4333061	0	0.020098195
CELSR2	2	0.65400596	0.00057004	1515.024919	207.5063573	0.020246709
CENPA	2	0.81731015	0.002580841	31015.8649	224.3198928	0.020352684
CENPE	3	0.93520758	0.001159387	6257.520811	994.9075733	0.020284964
CENPF	4	0.95281982	0.000561466	1471.449223	4374.163236	0.020221285
CEP250	2	0.77057412	0.000288459	391.3871077	346.6970753	0.020220806
CEP55	5	1.08432109	0.003948858	72711.36435	929.1763838	0.020493673
CEP76	5	0.916964	0.000715489	2387.594909	7353.185994	0.020245748
CFDP1	1	0.59914918	0.00745681	258503.9824	0	0.020517813
CHAD	1	0.56845266	8.09592E-05	37.45131098	0	0.019988056
CHEK1	17	1.39569198	0.049958249	11610101.95	5163.230453	0.020717779
CHGB	5	1.07148384	0.002043446	19422.65399	1169.984177	0.020360938
CHMP1A	2	0.5959174	0.004226549	83060.09263	3322	0.020428924
CHMP4B	3	0.98243216	0.006794263	214563.6994	123.4236966	0.020496134
CHN1	1	0.57083629	0.000841223	3295.020352	0	0.020255838
CIRBP	2	0.57718269	0.001984275	18304.42769	3322	0.020330868
CISH	3	0.96695853	0.002877341	39509.2049	18.91295756	0.020305716
CITED2	6	1.17877651	0.024991777	2903925.097	1082.818554	0.020590825
CKAP4	1	0.53127382	3.59713E-05	9.015894258	0	0.019917855
CKAP5	3	0.81415307	0.001828119	15580.98729	3322	0.020292922
CKS1B	4	1.02200795	0.007388278	254192.2818	1082.156588	0.020451931
CKS2	2	0.79607786	0.000861521	3459.597631	163.4614367	0.020268104
CLCA2	1	0.54810721	0.000480007	1097.549272	0	0.020168246
CLCN3	2	0.78452191	0.001292799	7837.988245	0	0.020316591
CLDN4	2	0.51412129	0.000669954	2147.426651	3322	0.020180167
CLEC4A	1	0.58869782	0.002748231	35675.41473	0	0.020371144
CLK1	5	1.05608307	0.002598582	31406.06257	5030.789437	0.020411336
CLK2	2	0.46189493	2.88014E-05	6.208682936	3322	0.019813128
CLK3	1	0.31613485	1.29389E-06	1.601853041	0	0.019438371
CLNS1A	2	0.84327163	0.002492874	28915.54127	254.6471779	0.020435038
CLU	7	1.17320565	0.006374859	188892.8182	9461.218906	0.020553883
CLUAP1	1	0.54037169	0.000286388	383.3650372	0	0.020150866

CMBL	2	0.81748607	0.000469399	1029.554424	177.563076	0.020177066
CNN3	1	1.11023275	1.04668E-17	1.543080635	0	0.000585823
CNNM4	1	0.3283395	2.11259E-06	1.669998255	0	0.019552145
CNOT1	3	0.95376738	0.005644849	148137.6529	545.9555155	0.020379899
CNOT7	6	1.121712	0.009621555	430726.3438	4546.535471	0.020490967
CNPY2	1	1.11023275	-6.72587E-17	1.543080635	0	0.000585823
COASY	1	0.49839827	0.000419611	833.6713558	0	0.020140642
COIL	12	1.29294662	0.013443275	840503.9576	31538.01988	0.020574197
COL14A1	2	0.84145879	0.001693699	13640.84524	23.5816883	0.020334743
COL18A1	4	1.02988176	0.000910608	3942.584284	1707.320837	0.020307165
COL1A1	20	1.38788808	0.006917646	227171.2429	26979.28268	0.020550914
COL1A2	9	1.25979527	0.0027921	37339.5743	3716.343503	0.020421836
COL2A1	14	1.31414875	0.001802112	15753.62966	10814.4226	0.020384524
COL3A1	4	1.03614241	0.000695667	2367.845492	370.6971528	0.020241667
COL4A5	4	1.03308112	0.001180449	6733.513767	1039.518062	0.020279903
COL5A1	5	1.10422288	0.000697204	2419.15074	832.8080765	0.020255117
COL5A2	1	0.48966186	1.83682E-05	3.749595202	0	0.019787404
COL6A1	3	0.83009046	0.000534495	1386.514045	3332.558297	0.02023303
COL6A2	2	0.76547943	0.000584914	1647.773679	3022.997893	0.020267623
COL6A3	1	0.57949384	0.000140387	102.3762505	0	0.020086607
COL9A3	2	0.84620701	0.006787933	216225.8341	1486.350858	0.020548687
COMP	2	0.83369765	0.000662414	2130.642824	0	0.020272196
COPS4	6	1.18056086	0.015820766	1165453.709	544.389676	0.020586604
COPS5	23	1.38950503	0.044324885	9134126.734	26267.89198	0.020713003
COPS7A	9	1.26463499	0.017136817	1367084.866	2263.349707	0.020613204
COPS8	9	1.27174832	0.017370601	1404974.433	4196.599045	0.020604744
COQ6	1	0.59132727	0.003038332	42932.03277	0	0.020387445
CORO1A	3	0.93056761	0.001543192	11106.39838	5971.128227	0.020420859
COX4I1	2	0.72359792	0.000237493	265.3253711	139.1498979	0.020050979
COX5A	2	0.77238868	0.000242603	277.0466854	526.8752598	0.020105297
COX6C	1	0.54620179	0.000223071	233.6846923	0	0.020030509
CP	1	0.30853584	4.18429E-07	1.593542651	0	0.019407431
CPNE1	10	1.04946038	0.003861447	69365.88385	21300.12332	0.020502534
CPNE3	2	0.4976436	8.77924E-05	38.60224105	3322	0.019922504
CRABP2	1	0.57524648	0.00129956	7863.663407	0	0.020227515
CREBBP	68	1.49317366	0.188493669	165147649.9	107782.3232	0.020888399
CREG1	3	0.96809808	0.00915963	390322.8079	1322.122104	0.020468853
CRIP2	1	0.54541747	0.000500726	1172.785887	0	0.020198076
CRKL	17	1.38161058	0.023109315	2516155.204	10122.60838	0.02064911
CRTC2	1	0.59087769	0.00238292	26408.51754	0	0.020401578
CRY2	3	0.73797715	0.0005005	1172.86178	136.1491268	0.02009441
CRYAB	3	0.95125258	0.002496239	28965.68957	332.5786547	0.020422569
CSE1L	9	1.21622737	0.01199209	668633.5335	8528.56909	0.020597285
CSF1	1	0.56003815	0.000434789	894.470175	0	0.020181122
CSK	13	1.35557426	0.043294858	8744107.768	6810.755866	0.020748502
CSNK1A1	11	1.32799108	0.030150436	4225264.834	8905.282498	0.020751529
CSRP2	1	0.58043325	0.000529605	1309.398852	0	0.020228953
CST3	5	0.94636348	0.000238229	275.1530629	3804.170096	0.02012592
CSTB	4	0.89987558	0.000235284	267.0834862	3379.81177	0.020125682

CTDSP1	1	0.45419586	8.21751E-05	33.23464615	0	0.019915763
CTDSP2	1	0.59936649	0.009529691	422045.606	0	0.020541763
CTGF	7	1.20461454	0.001728508	14177.55105	1343.688366	0.020368713
CTNNA1	10	1.26854194	0.014921529	1038475.055	3760.665708	0.020558091
CTNNB1	62	1.48813865	0.142298018	94158496.8	140893.4897	0.020910149
CTNND2	4	1.08352009	0.011023315	566105.205	99.93542256	0.020545225
CTSA	2	0.35428907	5.48655E-05	16.24952991	3322	0.01994089
CTSB	10	1.23881983	0.001049183	5153.243017	8679.561345	0.02036847
CTSC	1	0.55230304	0.000176973	148.4977548	0	0.020063705
CTSF	1	0.49054094	9.09256E-05	40.73059702	0	0.0200338
CTSH	1	0.47405108	1.05701E-05	2.340211299	0	0.019739124
CTSK	2	0.82966474	0.000713447	2387.176711	456.621106	0.020260406
CTSL1	10	1.23093948	0.003939321	72240.48274	18281.63391	0.020463209
CTSS	2	0.72929383	6.83703E-05	25.33974543	76.93180269	0.019974724
CTTN	7	1.21557651	0.014185822	938596.2347	2200.439481	0.020604993
CUL1	19	1.3915532	0.025889342	3117197.282	14557.47421	0.020658607
CUL2	14	1.29026224	0.008040669	300639.8718	15953.3408	0.020517567
CUL4B	8	1.24040682	0.024364367	2758760.583	2896.229215	0.020628648
CUL5	9	1.25145976	0.005810719	157246.2965	1059.468216	0.020400115
CX3CL1	1	1.11023275	1.14834E-17	1.543080635	0	0.000585823
CX3CR1	1	1.11023275	-1.14834E-17	1.543080635	0	0.000585823
CXCL1	1	0.56775676	0.000140475	103.6566357	0	0.020021112
CXCL10	3	0.91245181	0.000108549	62.77123145	98.41104932	0.020017355
CXCL12	4	1.07713855	0.001986985	18705.31844	968.1205267	0.020403041
CXCL6	1	0.56775676	0.000140475	103.6566357	0	0.020021112
CXCR4	12	1.34095529	0.026463463	3272118.613	6214.966717	0.020668862
CXXC5	2	0.85267833	0.008688114	350926.0482	0	0.020549182
CYB5A	1	0.37232554	0.000186715	163.8405245	0	0.020047447
CYBA	1	0.5743027	0.001001983	4673.561288	0	0.020310065
Cap1	1	0.47867268	8.4057E-05	35.07901903	0	0.020002812
Cct4	1	0.59260624	0.001415799	9332.977794	0	0.020359724
Cct5	1	0.59260624	0.001415799	9332.977794	0	0.020359724
Cct6a	1	0.59260624	0.001415799	9332.977794	0	0.020359724
Cct7	1	0.59260624	0.001415799	9332.977794	0	0.020359724
Cenpf	1	0.5125358	0.000173474	142.4672321	0	0.020101508
Chek1	1	0.59163149	0.002746897	35234.63826	0	0.020304991
Chn1	1	0.57083629	0.000841223	3295.020352	0	0.020255838
Cnot7	1	0.48228663	0.000120016	68.72354518	0	0.019970051
Cpne1	1	0.5125358	0.000173474	142.4672321	0	0.020101508
Ctnnb1	3	0.98851305	0.00689891	223998.0498	26.34782723	0.020477939
DACH1	4	1.10016728	0.022805668	2418315.889	297.8621654	0.02061171
DAP3	7	1.24038038	0.027018704	3392572.362	1418.344367	0.020629147
DAPK1	3	0.85405011	0.011942587	663693.0304	3580.276531	0.020586852
DAPK3	3	0.98870217	0.007763062	280137.4977	101.5936896	0.0205225
DAZAP2	11	1.29577145	0.01094249	557448.2849	12618.55486	0.020609719
DBF4	7	1.21356324	0.004735054	104921.6298	267.9823259	0.020383063
DCAF10	1	0.57566209	0.00087271	3551.188838	0	0.020185179
DCAF13	1	0.57566209	0.00087271	3551.188838	0	0.020185179
DCK	1	0.44687465	0.00011665	65.25714786	0	0.019985014

DCN	17	1.36468636	0.010071475	477099.0514	28581.42288	0.020619927
DCTD	1	0.47875113	3.21431E-05	6.597342383	0	0.019854609
DCUN1D1	6	1.18435753	0.003352846	52302.41535	1461.137176	0.0204128
DDB1	17	1.35333084	0.019426082	1755422.625	11590.10817	0.020589583
DDB2	6	1.19557914	0.015431095	1108051.007	494.4151554	0.020537068
DDIT3	9	1.26190184	0.018241705	1546638.329	2437.620389	0.020562549
DDIT4	1	0.58630756	0.003062441	43593.86637	0	0.020392316
DDOST	2	0.72112725	0.000521906	1273.938791	239.1690832	0.020106481
DDR2	4	1.07422346	0.010168744	485895.9995	3222.048227	0.020555616
DDX17	5	1.15580388	0.023605381	2589687.318	308.1361001	0.020608226
DDX39	2	0.57140937	0.001797107	15035.9529	3322	0.020297507
DDX5	13	1.35761217	0.060537079	17036409.77	10782.71828	0.020771983
DDX58	2	0.77924662	0.001194406	6638.210391	80.87468116	0.020237107
DEFB1	1	0.58690834	0.001236071	7108.870307	0	0.020330626
DEGS1	2	0.77510062	0.006733065	212685.8371	1765.742676	0.020535832
DERL1	2	0.69435817	6.31367E-05	21.58645614	4.531585857	0.019889773
DFFA	3	0.93909124	0.006386493	189644.4785	3689.031527	0.020513868
DGKA	1	0.59903146	0.006096127	173785.2979	0	0.020485311
DHX34	1	0.55666113	0.000250844	294.8671997	0	0.020142544
DHX58	1	0.55174074	0.000295223	408.0355893	0	0.020149201
DIABLO	3	0.91619348	0.000700722	2289.022339	153.429755	0.020196403
DKC1	2	0.73099365	0.000771381	2771.9438	256.9512873	0.020278698
DKK1	3	0.7697692	0.00021268	214.9986701	6885.92817	0.020151817
DLC1	1	0.59667537	0.005031206	117691.5856	0	0.020466154
DNAJA1	4	1.03980993	0.003870915	69659.16894	881.9350776	0.020458795
DNAJC1	1	0.47688311	0.000143514	99.89733139	0	0.020014774
DNAJC7	3	0.83653348	0.001475257	10133.7952	1111.970362	0.020299438
DNMT1	9	1.28963635	0.028035081	3657015.249	7515.236044	0.020640122
DOCK1	2	0.59946308	0.006108455	174494.4438	3322	0.020485803
DOCK2	3	0.97340294	0.003521777	58542.93176	169.8503724	0.020414265
DPF2	1	0.56755042	0.001023046	4874.788302	0	0.020146347
DPM1	3	0.82234752	0.00308757	44334.28045	12308.47207	0.020405724
DPM2	2	0.45211235	0.000138989	92.32335566	6640	0.020009378
DPP3	1	0.58966018	0.002579126	30925.80308	0	0.020424279
DPT	4	0.97987929	0.001215258	6933.348579	4070.398665	0.020375521
DRAP1	3	0.90763347	0.002241607	23404.12028	12.42907375	0.020313448
DSC2	1	0.57956252	0.001544364	11197.22201	0	0.020295335
DSCC1	1	0.59469486	0.003589563	60056.60715	0	0.020357539
DST	2	0.66562422	0.000483975	1116.158485	1140.788613	0.020173251
DTL	3	0.97819892	0.006629692	204442.493	14.80676562	0.020446787
DTX3L	3	0.81300911	0.004253649	84131.17335	5994.543575	0.020438708
DUSP1	3	0.96776425	0.006402537	190529.7352	314.5839353	0.020484574
DUSP10	1	0.59233201	0.00300818	42118.35613	0	0.02040109
DUSP4	2	0.7425008	0.003079675	44142.05427	302.6230589	0.020401578
DUSP6	2	0.83866115	0.003151102	46209.86113	1024.019134	0.020424768
DUSP9	1	0.59233201	0.00300818	42118.35613	0	0.02040109
DYNLL1	41	1.42527737	0.019753503	1814846.881	102407.2006	0.020727087
DYNLRB1	1	0.53688957	0.000763449	2719.770769	0	0.020206205
DYNLT1	2	0.26589711	1.22629E-06	2.236251533	3322	0.019411183

Dnmt1	1	0.59832978	0.006624797	204244.3044	0	0.02045904
E2F1	28	1.44156117	0.086390542	34724111.59	18703.27886	0.020769708
E2F2	3	0.97942257	0.009248623	397943.2909	13.59108298	0.020470817
E2F3	7	1.22744189	0.022318378	2317090.662	778.1580049	0.020583873
E2F4	8	1.26883137	0.036625067	6239151.392	576.6537451	0.020662357
E2F5	5	1.15856594	0.029558422	4062510.319	140.8212194	0.020618681
E2F7	1	0.57286999	0.000746032	2592.084588	0	0.020264254
E4F1	5	1.1644906	0.026170187	3187747.964	159.6693789	0.020639872
EBNA1BP2	1	0.54619884	0.000505872	1193.523961	0	0.020149677
ECT2	1	0.58271549	0.001291238	7761.157645	0	0.020323364
EDF1	2	0.83166317	0.003747866	65313.79845	0	0.020369442
EDN1	4	0.86259579	0.001124827	5922.630317	3862.722215	0.020316108
EDN2	1	0.53249354	0.000506927	1233.375263	0	0.020141355
EDNRA	1	0.46349854	5.05324E-05	13.77564084	0	0.019922272
EEF1A1	25	1.37999858	0.01178874	646106.6022	46752.1733	0.020635132
EEF2	2	0.8478877	0.011830492	651405.481	750.1075961	0.020584369
EFEMP1	1	0.52905297	2.60658E-05	5.668456081	0	0.01983569
EFNA1	1	0.51380379	0.000668602	2137.868756	0	0.02017969
EFNA3	1	0.51380379	0.000668602	2137.868756	0	0.02017969
EGFR	91	1.50183459	0.149293869	104048513.5	292001.0855	0.020952241
EGR1	9	1.27462867	0.043723227	8887432.318	5702.513359	0.020706219
EHD1	1	0.58480244	0.002588636	31520.12949	0	0.020362153
EIF2AK2	13	1.34592567	0.038186065	6778016.099	10651.34062	0.020748754
EIF2B1	5	1.10985412	0.009724558	439557.3315	1568.242181	0.020524721
EIF2C2	3	0.83544519	0.011238384	588035.937	7560.540483	0.020574445
EIF2S1	6	1.17241981	0.006899287	221317.8289	1642.590109	0.020534844
EIF3G	3	0.93392365	0.001875518	16354.14026	811.3051446	0.020393291
EIF3H	4	0.97279386	0.000778972	2827.780875	367.8671038	0.020243827
EIF3I	5	1.1265719	0.011724944	639092.2229	5219.028855	0.020607729
EIF4A1	3	0.83483442	0.00037376	654.278134	422.502882	0.020171821
EIF4A3	2	0.70343962	0.000520169	1262.480562	219.4517629	0.020155625
EIF4ENIF1	2	0.7506363	0.007572328	266578.7248	529.9812214	0.02051954
EIF4G1	9	1.21073859	0.002933861	40025.39186	6144.293999	0.020428924
EIF4G2	3	0.94513938	0.005237247	127518.8657	2871.547531	0.020499334
EIF6	17	1.31074191	0.004823963	108318.0486	27350.7987	0.02054028
ELF1	3	0.98964725	0.011617987	627538.8318	59.82778257	0.020504997
ELF2	3	0.9609374	0.004281312	85190.13983	296.0098235	0.020420859
ELF3	10	1.31622215	0.041677258	8075484.434	8307.038078	0.020731618
ELF4	2	0.84726276	0.006949351	224596.8263	16.75152062	0.020451196
ELL	1	0.60238036	0.011145498	578362.3686	0	0.020555863
ELMO2	1	0.37504144	0.00027442	356.2084747	0	0.020085425
EMG1	2	0.75064564	0.000181577	156.7060709	125.2011813	0.020113825
EN1	1	0.59603049	0.006371527	188713.1298	0	0.0204128
ENO1	3	0.97009506	0.004095055	77960.41466	771.9423373	0.020458304
ENY2	1	0.57432242	0.001709305	13586.84105	0	0.02027629
EP300	78	1.49841387	0.213875637	212607540.6	133341.6644	0.020938876
EPB41	15	1.33262479	0.02397513	2672696.984	29646.05555	0.020719791
EPHA2	7	1.05479037	0.01488275	1043284.783	15590.45443	0.020583873
EPN3	1	0.54729064	0.000109237	57.68273217	0	0.020045093

EPOR	13	1.35267301	0.026764278	3380193.063	2337.866737	0.020615444
EPS15	8	1.18444887	0.011574951	626269.3826	21843.51217	0.020627402
EPS15L1	1	0.35247336	2.35031E-05	4.341577847	0	0.019832694
EPS8L2	1	0.57479591	0.000424853	845.8909859	0	0.020237587
ERBB2	25	1.4357743	0.068367002	21840065.83	21246.76256	0.020805402
ERBB3	14	1.34340163	0.028319632	3766252.893	13131.22478	0.020639872
ERBB4	11	1.31901766	0.019125148	1723312.74	4631.520806	0.020607231
ERCC3	3	0.98932973	0.016103465	1207051.553	0	0.020572957
ERCC6L	2	0.5831239	0.001293849	7793.416947	3322	0.020323848
EREG	2	0.84576544	0.007566165	269003.3306	0	0.020537809
ERI3	1	0.54903297	0.000189579	170.7017556	0	0.020082117
ERO1L	2	0.79572753	0.000502732	1181.782422	123.3832102	0.020209554
ESR1	72	1.49709461	0.220812968	226600585.8	136568.5049	0.020968198
ETV6	7	1.21684722	0.010768484	539044.7436	2320.109682	0.020555121
EVI5	1	0.45384849	7.92297E-05	30.9882615	0	0.019904384
EVL	4	1.0622345	0.009650896	437000.2756	4629.893732	0.020549677
EXO1	3	0.9835704	0.005663828	149729.3555	0.513095238	0.020363853
EXOSC4	4	0.99428886	0.00173615	14019.08544	643.4722269	0.020385741
EXT1	1	0.50075898	0.000430071	861.6341495	0	0.020165387
EZH2	12	1.27705531	0.020433492	1940459.853	17534.53175	0.020653857
EZR	30	1.4379196	0.035869815	6020743.323	37341.00646	0.020780327
Eps15	1	0.35247336	2.35031E-05	4.341577847	0	0.019832694
Eps15l1	1	0.35247336	2.35031E-05	4.341577847	0	0.019832694
Esr1	1	0.57064942	0.0015315	10906.86039	0	0.020275567
F13A1	1	0.58606855	0.000581455	1627.332309	0	0.020256319
F2R	2	0.79945021	0.000606298	1750.771586	106.1757659	0.02023183
F3	1	0.57964904	0.001406361	9204.44148	0	0.020324816
FADD	17	1.37945151	0.017206288	1379728.037	6667.811701	0.020637876
FAM175B	1	0.58421517	0.001002374	4683.027446	0	0.020306199
FAM84B	1	0.5597477	0.000670344	2108.973072	0	0.020154911
FANCA	25	1.39123334	0.032906206	5033845.277	47844.56131	0.020690909
FANCG	5	1.0606351	0.003930442	71877.8766	4759.72514	0.020428679
FANCI	5	1.09021284	0.003511249	57429.21475	9839.584979	0.020429657
FAP	1	0.54206686	0.000229422	251.0346456	0	0.020082353
FAR1	1	0.46205066	7.56693E-06	1.987284499	0	0.019682906
FAS	15	1.37028199	0.024128309	2720120.625	14448.6502	0.020708982
FASN	1	0.56992061	0.000684994	2187.445381	0	0.020224639
FBLIM1	3	0.80046013	0.001546143	11131.14097	3322	0.020334501
FBLN1	6	1.18001668	0.002153191	21713.86758	1815.726596	0.020423791
FBLN5	2	0.4614272	2.88946E-06	2.388035172	3322	0.019625427
FBN1	4	1.021616	0.000597285	1730.848586	2018.467113	0.020255358
FBP1	3	0.96886462	0.003540253	58294.37936	911.2653554	0.020474745
FBXL2	1	0.56556466	0.000662416	2042.911324	0	0.020212426
FBXL5	1	0.56556466	0.000662416	2042.911324	0	0.020212426
FBXO32	1	0.56556466	0.000662416	2042.911324	0	0.020212426
FBXO5	3	0.82976126	0.001763613	14481.16141	3364.82223	0.020297507
FCER1A	2	0.74479961	0.002431358	27830.60715	83.13673352	0.020362153
FCER1G	2	0.74627826	0.002564423	30984.26496	167.242669	0.020387689
FCGRT	1	0.51300267	5.4631E-05	15.96385472	0	0.019963044

FCHSD2	1	0.5030677	1.95315E-05	4.052978806	0	0.019794058
FDPS	1	0.56315191	0.000838623	3287.980823	0	0.020247669
FDXR	1	0.46286989	0.000464296	1006.930253	0	0.020167769
FEN1	9	1.29735034	0.028326915	3733663.867	1671.998819	0.020632887
FERMT2	1	0.44494	6.94599E-05	24.22135241	0	0.019939958
FES	7	1.24319809	0.022078159	2276199.679	370.9494275	0.020616938
FGF18	4	1.02756467	0.00219511	22517.01662	142.6993306	0.020319493
FGF8	4	1.02756467	0.00219511	22517.01662	142.6993306	0.020319493
FGF9	4	1.02756467	0.00219511	22517.01662	142.6993306	0.020319493
FGFBP1	2	0.78571322	0.000106163	58.24408551	513.5889614	0.020113588
FGFR1	11	1.28616167	0.01866733	1623447.352	21485.74457	0.020654607
FGFR1OP2	3	0.94474748	0.00136758	8740.650166	0	0.020284
FGFR2	5	1.09246837	0.005186198	126942.3754	1297.277467	0.020475728
FGFR3	13	1.30405333	0.013622295	863220.317	14135.78474	0.020588838
FGFR4	5	1.09767094	0.01138622	603346.4076	1433.23018	0.020508446
FHIT	3	0.86042099	0.010335004	497158.881	3601.084681	0.02057147
FHL1	1	0.52184779	0.000490942	1125.171972	0	0.020105297
FIBP	5	1.01321224	0.000895154	3735.72586	6165.304399	0.020354869
FKBP4	6	1.20232804	0.022284683	2312628.383	3058.777012	0.020670614
FLI1	2	0.83462966	0.009817672	448198.39	99.76996293	0.020513129
FLNA	17	1.37557397	0.031304884	4556303.907	27915.57902	0.020734892
FLNB	5	1.10087438	0.003042022	43146.87611	1426.047178	0.020469589
FLOT2	1	0.58968641	0.00253384	30102.48911	0	0.020366526
FLT1	8	1.24418636	0.022502693	2360905.843	8962.418808	0.020633635
FMNL1	1	0.59213255	0.004147801	79987.71409	0	0.020445807
FMOD	3	0.92503254	0.000354222	615.3194552	22.56166774	0.020102929
FN1	27	1.41228486	0.012942892	789140.6259	55517.16332	0.020663608
FNTA	6	0.95092573	0.00104253	5078.161427	18458.94815	0.020324574
FOLR1	1	0.59226569	0.002455195	28387.0582	0	0.020384037
FOS	20	1.40209027	0.060339852	16924157.92	9418.227091	0.020707224
FOSB	1	0.55732023	0.00170429	13503.85026	0	0.020280867
FOSL2	2	0.83616575	0.008075818	303164.4437	8.552743793	0.020421592
FOXA1	1	0.59936649	0.009529691	422045.606	0	0.020541763
FOXM1	4	1.10139924	0.022237663	2298092.947	292.9393611	0.020630892
FOXP1	1	0.56931093	0.002025785	19100.26152	0	0.020320461
FSCN1	2	0.78549664	0.006442963	193197.0368	1297.484698	0.020498349
FST	1	0.58606855	0.000581455	1627.332309	0	0.020256319
FUNDC2	3	0.82191147	0.001130279	5946.533227	3409.577256	0.020313932
FXR1	2	0.7611776	0.00014817	105.4359092	30.26877828	0.02005734
FXYD6	1	0.60238036	0.011145498	578362.3686	0	0.020555863
FZD1	1	1.11023275	-4.86317E-17	1.543080635	0	0.000585823
FZD6	1	1.11023275	-1.03846E-17	1.543080635	0	0.000585823
FZD7	1	0.1959828	5.60054E-09	1.590637667	0	0.018783424
FZD8	1	0.30355334	4.30104E-07	1.593571878	0	0.019391778
Fkbp4	1	0.57083629	0.000841223	3295.020352	0	0.020255838
Flna	3	0.80557065	0.001563838	11407.30118	3352.233969	0.02034662
GAB2	13	1.35545112	0.031543056	4674124.514	1669.063909	0.020659357
GABARAP	3	0.37179844	4.011E-05	10.69177984	9958	0.01992669
GADD45A	13	1.3334411	0.032345153	4862270.475	9920.343892	0.020673368

GALNT10	1	0.56426034	0.001292309	7886.571232	0	0.020271955
GALNT6	1	0.58606855	0.000581455	1627.332309	0	0.020256319
GAPDH	10	1.24923702	0.020626061	1978690.312	14007.39088	0.02071426
GARS	2	0.71692862	0.001266851	7613.83218	822.4481717	0.020319977
GAS6	1	0.53790891	0.000668705	2132.880183	0	0.020150628
GATA3	4	1.03631372	0.004195532	81861.61891	286.7417157	0.020402065
GBP1	1	0.48784785	4.68353E-05	12.24046996	0	0.019930413
GBP2	4	1.0015521	0.001803457	15150.83971	1707.517246	0.020318768
GCH1	3	0.74728331	0.002892108	38904.72332	3396.148083	0.020425257
GEMIN4	10	1.20552397	0.001562714	11380.4852	13824.32139	0.020419637
GFRA1	2	0.68126314	0.001201827	6813.719128	0	0.020226077
GGA2	7	1.09054809	0.002110923	20800.56669	7403.379493	0.020419149
GJA1	4	1.06498426	0.010614274	527320.8804	2827.1476	0.020535585
GLB1	1	0.26172759	2.46481E-06	1.619171146	0	0.019561331
GLI3	4	1.04500719	0.01653532	1271237.562	2550.526987	0.02057767
GLRX	1	0.37541262	0.000381964	680.6673097	0	0.020073379
GLRX3	1	0.57809071	0.001050798	5186.761959	0	0.020303059
GLUD1	1	0.58829399	0.000887419	3676.462767	0	0.020317317
GLUD2	1	0.51991616	0.000324104	497.1164428	0	0.020145634
GLUL	3	0.95046083	0.002154129	21587.1867	2864.115867	0.020434793
GMFB	1	0.59233201	0.00300818	42118.35613	0	0.02040109
GMNN	4	1.08691128	0.008093324	304813.0346	315.2956532	0.020516334
GMPS	2	0.79649593	0.001080339	5442.334831	0	0.020313448
GNAS	4	0.89906796	0.001119097	5835.513823	3498.821119	0.020291957
GNAZ	1	1.11023275	7.95197E-17	1.543080635	0	0.000585823
GNB1	3	0.58694783	0.002468825	28368.09158	6642	0.020359967
GNB2	2	0.58281942	0.001481289	10212.02053	3322	0.020283036
GNB2L1	21	1.41258854	0.054732979	13929981.64	48667.8988	0.020770466
GNG11	1	0.37010424	0.000110911	59.02126297	0	0.019964445
GNG12	1	0.36845849	6.65464E-05	22.28990485	0	0.019890468
GNL3	1	0.60238036	0.011145498	578362.3686	0	0.020555863
GOLM1	6	0.80674345	0.002596562	31375.72038	13655.49997	0.020381359
GOSR1	1	0.46119558	0.000316754	471.8305736	0	0.020079518
GPBP1	1	0.60038967	0.009608287	429199.4929	0	0.020520773
GPC1	3	0.78338413	0.00039412	728.7547954	3433.487855	0.020199988
GPHN	2	0.58996262	0.000889221	3692.458204	13264	0.020319252
GPI	2	0.79005179	0.000625496	1842.625129	1255.06633	0.020265698
GPM6B	1	0.60093811	0.006706974	211028.0891	0	0.020533609
GPR183	1	0.56931093	0.002025785	19100.26152	0	0.020320461
GPSM2	1	0.52836997	0.000275551	354.9063447	0	0.020085188
GRB7	9	1.28621208	0.02226146	2333996.36	2594.913059	0.02058859
GRIPAP1	2	0.78429982	0.004050547	76299.28443	2924.491126	0.020427212
GRN	3	0.90440302	0.0001265	82.8280616	2573.521598	0.020098668
GSS	1	1.11023275	-4.17502E-17	1.543080635	0	0.000585823
GSTM4	4	1.07627428	0.014609511	993373.5962	3207.140567	0.020603998
GSTZ1	1	1.11023275	-2.62765E-18	1.543080635	0	0.000585823
GTF2IRD1	2	0.80300089	0.007534806	263938.5571	577.3062879	0.020526942
GTPBP4	2	0.84683144	0.011362213	600988.4759	2432.271175	0.020579158
Gab2	3	0.993858	0.008972119	381137.6431	0	0.020468362

Glud1	1	0.58829399	0.000887419	3676.462767	0	0.020317317
Glul	1	0.58829399	0.000887419	3676.462767	0	0.020317317
Gphn	1	0.58829399	0.000887419	3676.462767	0	0.020317317
H1F0	2	0.83833742	0.003665498	62509.64203	0	0.02040548
H2AFX	10	1.30980235	0.027228268	3452688.519	516.8472718	0.020577918
H2afx	2	0.841538	0.004520004	95345.88526	0	0.020340316
HAUS1	2	0.82859879	0.001578228	11652.18648	0	0.020258002
HCLS1	3	0.98036393	0.006796304	215335.9448	179.0042597	0.020515347
HDAC1	59	1.4856489	0.147464635	101119564.7	80680.52105	0.020874606
HDAC2	34	1.45716391	0.097177904	43913227.84	22072.69837	0.020805655
HEATR1	1	0.57453243	0.000594034	1651.332476	0	0.020242867
HERC5	5	1.15683337	0.014877567	1029914.324	27.10540742	0.020510417
HEXIM1	2	0.8632733	0.016626919	1287469.227	0	0.020641619
HGS	30	1.42495263	0.037248962	6464754.408	67974.95897	0.020782604
HIF1A	26	1.43486142	0.091792231	39162332.9	39748.07745	0.020811997
HINT1	1	0.4609791	0.000245519	282.1533679	0	0.020098905
HIPK1	3	0.99933727	0.014696045	1005045.975	1010.572583	0.020612208
HIPK2	13	1.3279363	0.039921957	7411801.913	11989.98188	0.020705968
HISPPD2A	1	0.5442772	0.000960571	4295.774097	0	0.020188282
HIST1H2AC	1	0.49019878	0.000244205	279.3443492	0	0.020057812
HIST2H2BE	6	1.14939198	0.008734317	354564.7922	731.589286	0.020526696
HJURP	1	0.82912642	1.29319E-18	1.589091778	0	0.000586166
HLA-B	10	1.27003468	0.009678191	438449.2408	11510.62606	0.020585362
HLA-C	2	0.71907742	0.000200486	195.0982997	0	0.020072435
HMGA1	7	1.23061403	0.025012122	2908269.917	594.861204	0.020577173
HMGB2	5	1.15850698	0.02723647	3449178.403	143.9663153	0.020667361
HMGCL	1	1.11023275	-6.3879E-17	1.543080635	0	0.000585823
HMMR	2	0.74113958	0.001315047	8186.921157	690.9452089	0.020338377
HN1L	2	0.59255428	0.004156189	80312.52178	3322	0.020446297
HNRNPA2B1	1	0.53839025	0.000192901	175.19945	0	0.020101272
HNRNPAB	2	0.84664756	0.002042818	19456.83482	111.2593153	0.020390855
HNRNPD	12	1.30958873	0.013148146	803539.4581	10592.81761	0.020596291
HNRNPL	7	1.16390563	0.004293887	85737.98845	5273.010302	0.020502288
HNRPDL	1	0.58823865	0.001673396	13089.82011	0	0.020370658
HOXB2	3	0.94515089	0.018211687	1542018.413	1276.438912	0.020566017
HOXB6	5	1.14995222	0.020172153	1891501.207	3510.972524	0.020609968
HPRT1	1	0.58241143	0.0014783	10170.02802	0	0.020282554
HPS5	1	0.53344724	4.55696E-05	12.17880318	0	0.019950678
HSD17B4	2	0.84235568	0.002100794	20541.98019	280.2823934	0.020408163
HSF1	14	1.36697744	0.045534343	9635593.107	6965.330688	0.020703457
HSP90AA2	1	0.52548049	0.000131141	82.12330847	0	0.020010785
HSP90B1	9	1.28613301	0.020059192	1872493.332	12579.96504	0.020658107
HSPA1L	1	0.57286999	0.000746032	2592.084588	0	0.020264254
HSPA4	22	1.41367356	0.0681684	21595929.9	25229.23851	0.020805148
HSPA8	27	1.433323	0.057410039	15317809.91	55776.02741	0.02083842
HSPB1	10	1.29284935	0.020252464	1906621.734	11067.5976	0.020672116
HSPB2	4	1.02145238	0.011337424	597353.189	7948.438995	0.020558339
HSPG2	12	1.30067398	0.001467976	10405.91832	9062.106638	0.020365068
HTRA1	5	1.01051804	0.000434761	951.4013892	4361.019836	0.020182792

HUWE1	8	1.25606005	0.018696667	1628526.041	5122.023328	0.020621421
HYOU1	1	0.52548049	0.000131141	82.12330847	0	0.020010785
Hdac1	1	0.55077005	0.001042721	5069.319593	0	0.020165149
Hipk1	1	0.60238036	0.011145498	578362.3686	0	0.020555863
ICAM1	6	1.18060795	0.009966619	466838.8557	1312.114306	0.020575933
ICAM2	3	0.97285917	0.002356286	26223.40266	77.82004781	0.020393291
ICAM3	4	1.07711542	0.005394618	135514.9723	802.0598174	0.020530646
ID1	4	1.04845382	0.005179567	124760.6331	20.52846116	0.020400846
ID3	8	1.21868957	0.011219152	585394.6644	1169.090767	0.020519787
ID4	2	0.80987508	0.002387413	26498.4361	0	0.020302093
IFI16	5	1.14256705	0.030745798	4395809.378	1396.173487	0.020667611
IFI27	1	0.58633199	0.003655652	62129.80962	0	0.02035317
IFI35	1	0.54112458	0.001024236	4879.470631	0	0.020224639
IFIT1	1	0.36862174	6.66812E-05	22.42494698	0	0.019890932
IFIT2	1	0.36862174	6.66812E-05	22.42494698	0	0.019890932
IFIT3	3	0.58322798	0.001484291	10254.3718	6642	0.020283518
IFITM1	1	0.53007814	0.000369558	645.9939879	0	0.020123784
IFNAR2	4	1.09552539	0.015991764	1190856.96	0	0.020549429
IFNGR1	4	1.08263204	0.010084512	475360.1242	1088.703349	0.020514854
IGF1	7	1.12829826	0.003423334	55114.30221	8745.71662	0.020445073
IGF1R	19	1.40495494	0.057621737	15519431.77	17580.00271	0.020773752
IGF2BP2	2	0.81939172	0.00071751	2398.007456	100.5747078	0.020276531
IGF2R	4	1.02354732	0.000817658	3118.610911	841.7071046	0.020273641
IGFBP2	1	0.53064387	0.000153792	114.6661207	0	0.02004627
IGFBP3	15	1.36763694	0.013199774	814526.9593	27056.04041	0.020654107
IGFBP4	1	0.53064387	0.000153792	114.6661207	0	0.02004627
IGFBP5	7	1.21939506	0.003462539	55935.37032	5510.540542	0.020516334
IGFBP7	5	1.07219483	0.000560877	1481.786391	1711.626941	0.020229433
IGHM	1	0.53265411	0.000351958	589.7062337	0	0.02011501
IGKC	1	0.37541262	0.000381964	680.6673097	0	0.020073379
IKBKB	18	1.38644663	0.037260131	6456606.395	27691.00779	0.020742703
IKBKE	10	1.1052073	0.002919138	39619.06894	18354.08954	0.020408163
IKZF1	8	1.22335054	0.023210435	2506569.523	5388.740128	0.020568743
IL15	2	0.72765196	0.000636017	1923.017354	0	0.020141831
IL15RA	3	0.85914777	0.001275971	7742.656953	63.10843838	0.020213623
IL16	1	0.59740928	0.003989151	74000.41531	0	0.020423302
IL1B	4	1.0541652	0.001032313	4975.572941	3304.081617	0.020398896
IL1R1	4	1.03090709	0.004596632	99582.22221	1679.809674	0.020473763
IL27RA	3	0.85465343	0.007050791	231876.8184	3322	0.020479658
IL2RG	9	1.23818904	0.012881438	778057.205	5822.394791	0.020544236
IL6	3	0.96854429	0.005191773	125336.3175	247.8587374	0.020472044
IL6ST	13	1.35780905	0.040338917	7606336.073	5085.550781	0.020687399
IL7R	5	1.1563972	0.016287574	1240922.947	291.2150152	0.020586852
IL8	4	1.02911734	0.00036724	652.584358	743.024064	0.020178736
ILF3	8	1.26100171	0.009874581	453204.1269	6089.024426	0.020577918
ILK	16	1.34710397	0.013222898	814475.5253	23194.98605	0.02064961
ING1	15	1.37687368	0.059377408	16399555.95	5153.236335	0.020740183
INPP5A	2	0.74250885	0.002425607	27363.4593	527.5146353	0.020416462
INPP5D	10	1.30749569	0.020628205	2011382.124	272.7442675	0.020567504

IQCG	1	0.56407474	0.000590586	1623.875339	0	0.020240947
IQGAP1	5	1.15118843	0.012037614	675104.7055	759.4939809	0.020575933
IRAK1	13	1.3349918	0.025861056	3109279.444	9826.200191	0.020659107
IRF1	6	1.19359732	0.021446524	2137726.804	625.3653119	0.020606734
IRF3	8	1.19177224	0.021381818	2125734.738	13402.65252	0.020592812
IRF5	3	0.92905734	0.001261101	7401.200395	162.974099	0.02024839
IRF7	7	1.23091765	0.024019218	2682173.303	1477.658623	0.02060176
IRF8	4	1.06600607	0.007321763	249216.1876	59.76691552	0.020432836
IRF9	2	0.85649602	0.008676344	349963.7722	24.74967822	0.020517813
IRS1	20	1.41349955	0.040317679	7597494.539	12843.7258	0.020743963
ISG15	5	1.06096033	0.00520506	125916.1556	2974.074693	0.020465172
ITGA3	7	1.14104835	0.001014356	4889.801357	5960.92952	0.02034565
ITGA5	12	1.32046013	0.012811292	771171.5744	12603.32462	0.020614199
ITGA6	5	1.08538973	0.000827826	3226.251775	1605.585068	0.020303059
ITGAV	11	1.27883145	0.002118361	21256.18013	7447.962423	0.020393534
ITGB2	6	1.1650432	0.004078269	77617.60268	1798.390867	0.020484819
ITGB4	8	1.21029037	0.010684704	536436.1344	13071.2088	0.020571966
ITGB5	8	1.22836147	0.004817058	108188.9369	4726.886342	0.020528177
ITGB6	5	1.13341985	0.002389407	26766.41727	917.0440117	0.020437729
ITM2C	1	0.82912642	-1.03187E-17	1.589091778	0	0.000586166
ITPKB	1	0.55209005	0.00102737	4920.289047	0	0.020259204
ITPR1	7	1.16004336	0.006588906	202070.0893	10374.3506	0.020562549
ITPR3	2	0.53790991	0.000296603	413.1241183	3322	0.020159671
Igfbp7	4	1.01506229	0.00015738	135.3526908	393.0031672	0.02004321
Ikbkb	1	0.57083629	0.000841223	3295.020352	0	0.020255838
JAG1	3	0.90187848	0.001556038	11267.94885	722.3117796	0.020340316
JAG2	1	0.56555156	0.001373362	8772.81149	0	0.020269307
JAK1	28	1.43802347	0.05436946	13796884.19	19408.36448	0.020729101
JAK2	26	1.43736425	0.065657612	20161901.27	13856.06309	0.020784123
JUN	45	1.47155356	0.141826992	93485008.25	42858.07047	0.02082647
JUNB	9	1.25614259	0.037936641	6688722.114	4909.334427	0.020689154
JUP	16	1.3750868	0.03437676	5517569.51	12136.63123	0.02070421
KANK1	1	0.58538039	0.002247375	23509.72524	0	0.020353655
KAT2A	15	1.34594999	0.038048258	6728639.496	17353.42881	0.02068439
KCNJ3	1	0.37010424	0.000110911	59.02126297	0	0.019964445
KCNK15	1	0.59087769	0.00238292	26408.51754	0	0.020401578
KCTD11	1	0.59832978	0.006624797	204244.3044	0	0.02045904
KCTD13	4	1.0222367	0.005036382	118160.4713	2064.956453	0.020436016
KCTD5	2	0.82064209	0.000772277	2783.10317	149.9520375	0.020238547
KDELR2	1	0.47536403	8.28671E-05	33.6620226	0	0.019929482
KDM2A	1	0.59914918	0.00745681	258503.9824	0	0.020517813
KIAA0101	1	0.59469486	0.003589563	60056.60715	0	0.020357539
KIAA0182	3	0.96757109	0.008557692	340582.3943	1154.783936	0.020529905
KIAA0528	1	0.50373453	4.02145E-05	9.356161991	0	0.019959543
KIAA0776	1	0.37541262	0.000381964	680.6673097	0	0.020073379
KIAA1279	3	0.90784868	0.000607865	1725.118787	795.3233253	0.020199032
KIAA1715	1	0.37365054	0.000189876	169.4436361	0	0.020030744
KIAA1967	3	0.97788377	0.004751312	104922.3728	618.3950451	0.02045904
KIF11	2	0.82149485	0.001896217	16740.99049	356.7851253	0.020344922

KIF15	2	0.74201365	0.001339009	8341.991075	207.7568441	0.020339831
KIF18A	1	0.58538039	0.002247375	23509.72524	0	0.020353655
KIF20A	1	0.53589814	0.000140259	94.01233906	0	0.01996935
KIF23	1	0.58271549	0.001291238	7761.157645	0	0.020323364
KIF2C	1	0.5793091	0.001583791	11691.85093	0	0.020275326
KIF5C	1	0.5848568	0.003663441	62388.94308	0	0.020353655
KIT	15	1.35656028	0.028379234	3786335.755	9512.271698	0.020651359
KLF10	1	0.58802703	0.002662828	33044.68502	0	0.020299438
KLF5	4	1.0985878	0.024654251	2825677.509	61.32432935	0.020576677
KLF6	3	0.9019605	0.011421951	607380.3864	3563.979557	0.020587845
KLHDC2	1	0.56139909	0.000917967	3919.800974	0	0.020246949
KLHDC5	1	0.56444885	0.000603934	1700.438235	0	0.020170391
KLHL24	1	0.58210833	0.00199128	18450.57455	0	0.020303784
KLHL9	1	0.58210833	0.00199128	18450.57455	0	0.020303784
KNTC1	1	1.11023275	2.46979E-17	1.543080635	0	0.000585823
KPNA1	9	1.27764471	0.015540429	1122848.297	3602.127711	0.020582136
KPNA2	14	1.34958038	0.048217333	10810458.33	11075.11188	0.020737915
KPNA3	4	1.06448131	0.006273212	182945.0685	4319.476349	0.020489983
KPNA4	2	0.82202873	0.002038091	19316.03668	0	0.020377467
KPNB1	23	1.40819443	0.033374798	5178059.601	35041.09523	0.020761877
KRT1	2	0.69031286	0.001482268	10225.46268	2258.337823	0.020285687
KRT10	2	0.86031001	0.012488016	724764.0085	0	0.020592067
KRT13	1	0.51622333	0.000331446	522.6352989	0	0.020173251
KRT15	10	1.21861868	0.002977738	41257.26806	6270.417567	0.02036021
KRT17	5	1.09426299	0.008450956	334361.0927	3855.842023	0.020583625
KRT19	9	1.19368773	0.002823325	37169.70975	9882.673983	0.020444338
KRT5	2	0.58486167	8.83328E-05	38.68875776	81.12643623	0.019962344
KRT6A	2	0.78814105	0.00051343	1237.432355	151.464208	0.020225837
KRT6B	2	0.78504619	0.000260611	321.1776395	0	0.020104586
KRT7	5	1.06503817	0.007377826	255375.6458	6311.471811	0.020577173
LAMA5	3	0.89888258	0.000251935	304.3750148	1047.83797	0.020205248
LAMB1	1	0.45394696	2.4012E-05	4.709797511	0	0.019842377
LAMP2	2	0.54778215	0.001218813	6908.629333	6640	0.020334985
LAPTM5	8	1.20631606	0.002431563	27522.63966	5395.259389	0.020443848
LARS	1	0.52548049	0.000131141	82.12330847	0	0.020010785
LASP1	5	1.14366295	0.005528646	142078.9217	5169.139559	0.020544236
LASS2	1	1.11023275	-3.55038E-17	1.543080635	0	0.000585823
LAT	4	1.08361631	0.008878677	372597.8015	5.919973016	0.020487524
LBR	2	0.79910561	0.000690603	2222.441533	190.5615385	0.020205009
LCP1	1	1.11023275	2.94188E-17	1.543080635	0	0.000585823
LCP2	9	1.28864261	0.018489894	1616630.744	619.8982252	0.020561806
LDHA	3	0.98518035	0.002163089	21775.25752	854.438991	0.020420126
LDLR	2	0.78761	7.98805E-05	33.44638882	93.71064156	0.020056869
LEPR	6	1.16839543	0.01129589	595780.3768	2209.180358	0.020525955
LGALS1	5	1.08443509	0.001045775	5211.831439	2102.211652	0.020287134
LGALS3	4	1.05116367	0.00161207	12095.94593	3151.309614	0.020442135
LIG1	2	0.84565833	0.005518153	141911.8892	7.739320542	0.020376007
LILRB1	4	0.97873595	0.003246658	49777.18347	2496.471632	0.020407431
LIMK2	1	0.31328859	2.32024E-05	4.115427554	0	0.019782359

LMNA	10	1.30369276	0.025825687	3100398.307	8043.852831	0.020686396
LMNB1	8	1.2404994	0.012491836	725455.7992	5762.388806	0.020584369
LMNB2	1	0.519459	0.000411252	789.2687323	0	0.020133991
LMO2	6	1.04067028	0.005488594	140214.6825	7437.612988	0.020494657
LMO4	3	0.98862174	0.01090476	552950.3565	349.7909247	0.020533115
LOX	1	0.49535691	5.45951E-05	16.08278165	0	0.0199794
LOXL1	1	0.31591567	1.29808E-07	1.594105469	0	0.019257671
LRIG1	1	0.58424583	0.002349467	25942.62748	0	0.020360453
LRP2	8	1.20059421	0.000650009	2008.785609	4814.409235	0.020336197
LRP6	2	0.43552085	9.5739E-06	2.742896693	3322	0.019764722
LRP8	5	1.04517113	0.00043425	886.5515054	1685.376234	0.020266179
LRPAP1	7	1.13943614	0.000432938	883.2937029	3628.555501	0.020250071
LRRC14	1	0.58210833	0.00199128	18450.57455	0	0.020303784
LSM1	7	0.96450877	0.001198177	6839.378477	10878.36504	0.020273159
LSM4	2	0.76075233	0.001078064	5408.907322	501.1979166	0.02024887
LTF	2	0.44585097	9.31402E-06	2.726590005	3322	0.019780984
LUC7L2	7	1.15615239	0.00149473	10405.10996	5471.896758	0.02043357
LUM	2	0.81240397	0.000256556	324.6350289	38.5453848	0.020122124
LXN	1	0.36619758	8.91429E-05	38.56682148	0	0.019936465
LYN	33	1.44881588	0.054651408	13967044.71	41583.38288	0.02079653
LYZ	3	0.80263824	0.000206907	205.6339841	6929.124317	0.020168723
Ldha	1	0.58829399	0.000887419	3676.462767	0	0.020317317
M6PR	1	0.5221434	9.48325E-05	44.11853903	0	0.020021347
MACROD1	1	0.57188237	0.001077074	5400.80811	0	0.020310306
MAD2L1	8	1.23115574	0.004788735	106661.8084	1852.328372	0.020423058
MADD	3	0.961511	0.001337703	8345.21717	173.6881768	0.020294369
MAF	7	1.22953765	0.030409495	4299509.16	163.9927901	0.020587845
MAFG	4	1.05890501	0.011456142	610204.848	717.4097586	0.020540775
MAGED2	1	0.58043325	0.000529605	1309.398852	0	0.020228953
MAGI3	2	0.24364833	1.24665E-07	2.228997601	3322	0.019133125
MALL	1	0.58968641	0.00253384	30102.48911	0	0.020366526
MAML2	2	0.84350183	0.009841373	450336.5646	53.16111823	0.020483099
MAOA	1	0.56992061	0.000684994	2187.445381	0	0.020224639
MAP1B	5	1.1397971	0.003299861	50673.15577	3908.21702	0.020477939
MAP2K1	12	1.32243848	0.024928935	2893455.759	27584.55904	0.020734892
MAP2K2	1	0.44651456	7.02549E-05	24.78395955	0	0.019951611
MAP2K4	6	1.12954383	0.009811052	447713.0809	2028.197078	0.020539292
MAP2K7	3	0.91220129	0.003583022	59760.19159	86.05831528	0.020411336
MAP3K2	3	0.91375565	0.002984644	41437.49351	231.6282036	0.020415486
MAPK14	36	1.44921281	0.066960567	20850007.37	48668.06326	0.020814281
MAPKAPK2	7	1.22523672	0.017144521	1368557.15	4038.360097	0.020613452
MAPRE1	5	1.12609912	0.004631542	99860.78065	317.5471971	0.020397921
MAPRE2	1	0.5104302	0.000246573	285.4081961	0	0.020093937
MAPT	14	1.36504577	0.02232186	2316476.512	14146.24772	0.020676623
MARCKS	2	0.78170128	0.000540618	1362.733023	202.3297592	0.020238307
MARS	3	0.97375226	0.003645207	61874.47966	2048.809384	0.020448991
MCF2	1	0.57572875	0.002885402	38700.98013	0	0.020393778
MCL1	9	1.24845317	0.009667647	434797.8382	3345.036792	0.020547698
MCM10	17	1.35395248	0.018975398	1676983.293	14723.24653	0.020554131

MCM2	19	1.39198253	0.019097749	1701340.366	4302.262082	0.020546461
MCM3	13	1.33480268	0.016155153	1214904.123	5757.139371	0.02061171
MCM4	12	1.31797111	0.011625894	631024.8536	1930.882278	0.020514361
MCM5	11	1.31560642	0.011866223	655587.7827	6323.525469	0.020580895
MCM6	13	1.33140438	0.008236441	317310.1989	2773.803222	0.020463209
MCM8	3	0.97390407	0.002027488	19324.28815	7.556278323	0.020238307
MDFI	14	1.28622551	0.003181386	47082.69781	16540.53985	0.020418172
MDH1	1	0.60093811	0.006706974	211028.0891	0	0.020533609
MDM4	14	1.37487718	0.059480407	16450475.94	8447.850891	0.020790451
ME2	1	0.58829399	0.000887419	3676.462767	0	0.020317317
MEAF6	2	0.82097244	0.001615632	12152.9678	49.15706897	0.020337166
MED1	13	1.34691451	0.052297972	12713744.67	9733.076041	0.020728849
MED21	6	1.161734	0.032992255	5059306.379	3376.309045	0.020650859
MED24	5	1.15426331	0.025639589	3055200.857	177.162431	0.020634134
MED8	3	0.95094215	0.003495926	56819.62383	155.6586448	0.020352927
MEF2A	4	1.09622775	0.018338213	1562826.401	632.2297049	0.020633635
MELK	3	0.96171951	0.002891448	38861.89116	75.00965892	0.020385497
MEN1	10	1.30371448	0.023084875	2476758.058	4609.899634	0.020672116
MET	19	1.40174663	0.052297942	12778759.57	12816.81763	0.020771983
METAP2	1	0.57431799	0.001715495	13687.78724	0	0.020338135
MFAP2	2	0.75724987	5.78095E-05	21.31105666	6.913783009	0.019925993
MFNG	2	0.75140411	0.001443266	9689.8694	0	0.020291716
MGRN1	3	0.90863766	0.004258478	84322.81342	4678.260984	0.020438953
MID1	4	1.03479551	0.001355701	8569.012562	1381.434349	0.020310065
MIF	5	1.10682427	0.013392617	834909.779	9040.638172	0.020604246
MKI67	2	0.74066809	0.002291341	24439.22238	217.3681636	0.020358996
MKL1	2	0.5959174	0.004226549	83060.09263	3322	0.020428924
MKL2	1	0.37365054	0.000189876	169.4436361	0	0.020030744
MKNK2	3	0.98948615	0.013059927	792900.6709	1099.642904	0.020584618
MLF2	1	0.45646934	3.45887E-06	1.776969541	0	0.019628586
MLLT6	1	0.51237669	0.000336288	528.1296306	0	0.020016886
MLPH	3	0.74987749	0.000242671	277.8403483	378.2925826	0.020098668
MLXIP	1	1.11023275	-6.09738E-17	1.543080635	0	0.000585823
MME	7	1.13669371	0.011283943	600524.43	9701.317358	0.020543988
MMP1	9	1.23740469	0.002402422	27433.48266	5771.287365	0.020413532
MMP12	1	0.54206686	0.000229422	251.0346456	0	0.020082353
MMP14	10	1.24865955	0.002918703	40076.68615	11681.27145	0.02044189
MMP17	1	0.57949384	0.000140387	102.3762505	0	0.020086607
MMP2	21	1.37470028	0.003124939	46568.03428	26050.18189	0.020487032
MMP3	9	1.22617741	0.001196565	6832.825018	3696.320174	0.020340801
MMP7	4	1.06936072	0.001942102	18019.75295	103.8118888	0.02034371
MMP8	2	0.77476284	8.2392E-05	35.56810758	75.36779487	0.020009143
MMP9	15	1.31043556	0.003126899	47047.90396	15464.05264	0.020418905
MNAT1	9	1.29289887	0.0468256	10195691.09	4164.909913	0.020729101
MOAP1	3	0.96717715	0.002699413	33908.66434	311.1573488	0.020416218
MOBKL1B	4	1.00946032	0.001174512	6423.020608	1650.232284	0.02034856
MORF4L2	6	1.16473899	0.01672915	1301904.594	849.2246922	0.020574197
MPHOSPH6	8	1.15691487	0.016993992	1344147.319	10770.75109	0.020611461
MPND	1	0.48176887	0.000119758	68.79610757	0	0.019984546

MPZL1	2	0.8030109	0.006174123	178235.9228	648.7289138	0.020504997
MRE11A	13	1.34982954	0.042929437	8575347.851	1798.902879	0.020656607
MRFAP1	2	0.82539883	0.007163566	238680.5042	0	0.020446542
MRFAP1L1	1	0.82912642	-3.07532E-17	1.589091778	0	0.000586166
MRPL42	1	0.58043325	0.000529605	1309.398852	0	0.020228953
MRPS14	1	0.49734612	8.76152E-05	37.80094307	0	0.019922039
MRPS31	2	0.7827232	0.00030433	434.737322	858.7386082	0.020185417
MRPS35	1	0.49734612	8.76152E-05	37.80094307	0	0.019922039
MS4A7	1	1.11023275	-3.71583E-17	1.543080635	0	0.000585823
MSH2	14	1.35975	0.038714545	6976855.94	3867.335405	0.020619677
MSH6	13	1.35608969	0.047835621	10647251.13	1248.209232	0.02064936
MSN	17	1.3763362	0.012501648	732732.0421	5524.253258	0.020605987
MST1	2	0.72301066	0.000196279	184.3679816	71.40018999	0.020090153
MSX2	1	0.59213255	0.004147801	79987.71409	0	0.020445807
MTA1	12	1.31874482	0.045092955	9455240.951	16681.16233	0.02073036
MTCH1	1	0.57769367	0.001291789	7798.523312	0	0.02030644
MUC1	10	1.2919579	0.028766139	3874786.873	4418.569259	0.02067988
MX1	4	1.09046636	0.006821648	216405.1631	1296.605127	0.020530893
MYB	12	1.33969719	0.039965971	7426271.637	1565.396897	0.020653108
MYBL2	10	1.31713635	0.041219584	7903293.312	658.1124357	0.020628399
MYCN	1	0.54112458	0.001024236	4879.470631	0	0.020224639
MYD88	7	1.18972704	0.003770513	66177.77115	1141.871825	0.020414753
MYH10	4	0.94564342	0.000861175	3464.178774	7564.094599	0.020280385
MYH9	7	1.20433655	0.006778486	214726.2216	5440.825088	0.020522007
MYL9	3	0.83561601	0.011473222	612686.8227	7379.763638	0.020573949
MYO10	2	0.82975166	0.001103824	5685.945724	466.0329883	0.02036604
MYO1B	1	0.5343439	4.29934E-05	10.73663905	0	0.019931576
MYST2	13	1.32585051	0.027798006	3594173.072	11000.58102	0.020695926
MYST3	2	0.83340004	0.004111447	78630.51804	6.79148134	0.02032167
MYST4	1	0.58137743	0.002319243	25018.55936	0	0.020268826
Map2k1	1	0.5125358	0.000173474	142.4672321	0	0.020101508
Mapk14	2	0.73165212	0.001591448	11776.5167	39.71538025	0.020266179
Mcm2	1	0.57577454	0.000852463	3401.762953	0	0.020151104
Mcm6	1	0.57577454	0.000852463	3401.762953	0	0.020151104
Mdh1	1	0.58829399	0.000887419	3676.462767	0	0.020317317
Mta1	2	0.84237948	0.001654935	12851.45486	209.6101071	0.020395484
Mybl2	2	0.85282065	0.008227416	314866.7185	8.53556003	0.020472044
Myh10	1	0.32757841	1.74156E-06	1.60893862	0	0.019510801
Myo10	1	0.58829399	0.000887419	3676.462767	0	0.020317317
N4BP1	1	0.49139813	5.38277E-05	16.06722859	0	0.01988097
NAB2	1	0.56092964	0.00196425	17947.62955	0	0.020297265
NBN	13	1.34735199	0.039468454	7249655.545	1995.570932	0.020630892
NBR1	1	0.52175695	0.000157802	117.8397699	0	0.020036622
NCAPD2	2	1.1455403	2.35072E-17	2.70827166	0	0.000586166
NCF1	13	1.34584169	0.022303636	2312792.338	10826.44331	0.02071954
NCK1	14	1.34651738	0.01708452	1374410.157	7176.502158	0.020604744
NCL	7	1.23683234	0.019913106	1845562.142	2263.214781	0.020619927
NCOA2	21	1.40526872	0.081546348	30905181.67	14165.20487	0.020764908
NCOA6	22	1.42766035	0.099445682	45960985.77	16105.40103	0.020791717

NDC80	10	1.23011619	0.009833463	449714.8381	9727.882911	0.020520527
NDRG1	17	1.32202076	0.015247628	1081195.769	24898.88897	0.020630643
NDUFA4L2	3	0.82109383	0.0009565	4271.426754	3767.306913	0.020342255
NDUFA6	1	0.56407474	0.000590586	1623.875339	0	0.020240947
NDUFAF3	1	0.49139813	5.38277E-05	16.06722859	0	0.01988097
NDUFS1	2	0.83174713	0.004301577	86053.59587	256.5327497	0.020445317
NEDD4L	5	1.10676765	0.0020477	19591.06652	1277.220735	0.020397433
NEDD9	6	1.20512709	0.014830296	1024043.07	1812.935779	0.020630643
NEK2	3	0.91608313	0.002702099	33986.03355	1319.506403	0.020385254
NEK3	2	0.81847029	0.00271619	34743.2379	0	0.020364582
NEK9	2	0.36890703	5.82435E-05	18.12316129	3322	0.019930646
NF1	2	0.73806118	0.000194604	181.9343742	253.8018417	0.020136841
NF2	10	1.31118327	0.023133586	2495970.872	5532.567388	0.020704461
NFATC1	5	1.15383651	0.018831781	1648175.345	623.830477	0.02060748
NFE2L1	3	0.9603165	0.008590481	343049.4345	37.29451634	0.020436261
NFIB	1	0.37541262	0.000381964	680.6673097	0	0.020073379
NFIC	3	0.60041194	0.008502331	336129.5564	6642	0.020473272
NFKB1	33	1.45106561	0.090870198	38375999.88	28504.59981	0.020784882
NFKB2	10	1.27217163	0.014447983	970372.1259	4176.202888	0.020553883
NFKBIE	5	1.1473558	0.008101232	305054.7421	569.9148701	0.020489491
NGFRAP1	6	1.13823498	0.001776385	14701.91342	1602.59863	0.020378926
NID1	8	1.22370478	0.002683363	33671.03715	6439.163353	0.020461492
NID2	3	0.98301451	0.001608025	12070.1318	630.6176491	0.020385254
NISCH	3	0.99532632	0.011916492	660169.8978	350.7441179	0.020603003
NKRF	2	0.83365033	0.004731382	104072.2793	0	0.020373332
NLK	6	1.20662455	0.026109049	3168117.469	717.0703895	0.020629895
NME1	5	1.0193019	0.00277574	35933.7956	5426.370665	0.020344437
NME3	1	0.5052352	0.000124699	74.62468954	0	0.019949513
NME4	2	0.77039563	0.000189482	170.1861339	156.7223491	0.020070311
NMI	8	1.17676034	0.022798979	2415924.906	10906.15288	0.020630643
NOLC1	4	1.08165103	0.008496722	335541.7301	1220.2833	0.020517074
NOS1AP	2	0.76474261	4.87099E-05	15.0790315	17.19590205	0.019942055
NOTCH1	12	1.2987472	0.030570343	4343986.08	13564.46286	0.020677124
NOVA1	1	0.56404125	0.001086953	5500.933048	0	0.02023303
NP	2	0.83192207	0.011348108	599611.4719	451.1058344	0.020559329
NPDC1	8	1.26080231	0.015976289	1187172.904	5964.629666	0.020580647
NPEPPS	1	0.56781426	0.000216715	221.8452422	0	0.020137791
NQO1	3	0.99923102	0.016875448	1325262.343	10.33543187	0.020594303
NR1D2	2	0.85090248	0.005381088	134651.2384	171.279308	0.020463209
NR1H3	9	1.26885678	0.015997307	1189526.775	1403.853513	0.020530646
NR2F2	3	0.86216281	0.016265912	1230386.58	3341.929209	0.020563787
NRAS	2	0.84231057	0.003030666	42705.11214	1132.741978	0.020453157
NRBF2	3	0.98467921	0.006531916	198359.4624	1.569425058	0.020388906
NRIP1	13	1.34079731	0.047773234	10607552.54	6169.324138	0.020669113
NRP1	7	1.048242	0.002297634	24677.87954	7832.11522	0.020355111
NRTN	2	0.68126314	0.001201827	6813.719128	0	0.020226077
NTRK1	7	1.23296945	0.013577315	867975.8733	4929.613969	0.020599025
NTRK2	5	1.14613753	0.009907742	463368.1143	2171.795349	0.020549677
NUDCD1	1	0.48176887	0.000119758	68.79610757	0	0.019984546

NUDT21	2	0.82185243	0.000358284	602.6891934	136.7226616	0.020183747
NUDT3	1	0.48540381	6.87548E-05	23.7595596	0	0.019974491
NUF2	4	1.0516517	0.003542611	58441.13522	280.5033731	0.020359967
NUMA1	7	1.11806715	0.006133635	174855.9998	15120.36249	0.020485557
NUP155	1	1.11023275	3.78048E-17	1.543080635	0	0.000585823
NUP205	2	0.78129613	0.001827206	15522.26217	0	0.020295093
NUP93	4	1.0145951	0.002105996	20621.12288	2031.889984	0.020353413
NUTF2	2	0.81877179	0.001566501	11418.49698	323.6063579	0.020379899
Ncoa2	3	0.99037134	0.008092539	304406.4681	29.10411987	0.020454137
Nfkb1	1	0.59104975	0.003881065	70177.80957	0	0.020358267
Nid1	1	0.58182435	0.000310773	476.8853551	0	0.020148012
Notch1	1	0.57432242	0.001709305	13586.84105	0	0.02027629
Nras	1	0.57083629	0.000841223	3295.020352	0	0.020255838
Nrip1	1	0.59832978	0.006624797	204244.3044	0	0.02045904
Nrp1	1	0.43545673	2.05107E-05	3.682426494	0	0.019788781
OAZ1	2	0.70271796	0.006720831	211910.5281	3637.775424	0.020534597
ODC1	2	0.58299865	0.000308445	453.0273107	410.0206802	0.020139454
OFD1	3	0.96548013	0.001029897	4941.373761	585.5431594	0.020342498
OPTN	1	0.58430552	0.002710746	34176.34103	0	0.020298231
ORC1L	11	1.31242547	0.013085512	799072.6077	760.0097473	0.020509925
ORC6L	9	1.26651661	0.006443914	194646.4628	161.7828376	0.020385497
ORMDL3	1	0.58043325	0.000529605	1309.398852	0	0.020228953
OS9	2	0.83974661	0.004340448	87580.24305	429.6627745	0.020437484
OTUB1	8	1.20102445	0.01126046	589724.0418	11284.54325	0.020552646
OTUD7B	1	1.11023275	-1.77603E-19	1.543080635	0	0.000585823
P4HB	10	1.29290372	0.00383794	68709.74942	10596.571	0.020519047
PAAF1	6	1.02256218	0.000519169	1279.469712	744.1219644	0.020201901
PAICS	5	1.09831453	0.001810997	15300.12067	4572.411191	0.020417683
PAK1	18	1.4034515	0.040371505	7602285.489	36400.35384	0.020791717
PALLD	3	0.97975033	0.002576193	31216.31314	384.8544089	0.020430635
PARK7	1	0.44156817	0.000135388	87.04405438	0	0.020019233
PARP1	18	1.39997617	0.05432613	13726259.57	23609.1546	0.020754809
PARP9	1	0.44879051	0.000191094	171.6772916	0	0.020040151
PARVA	2	0.83223773	0.001713958	13714.4609	240.3723397	0.020395727
PARVB	1	0.57453243	0.000594034	1651.332476	0	0.020242867
PAXIP1	1	0.59046506	0.004630108	99704.11342	0	0.020429168
PBK	2	0.85461223	0.012824296	765643.6073	32.69073296	0.020575933
PBX1	2	0.4867384	3.87662E-05	9.428299708	3322	0.019888383
PBXIP1	1	0.82912642	-2.45164E-17	1.589091778	0	0.000586166
PCGF2	3	0.90653086	0.001664566	12895.35396	869.3693324	0.020293646
PCM1	3	0.78541674	0.000261138	323.3498496	3322	0.02010506
PCNA	46	1.46343882	0.079901873	29707110.65	57960.02526	0.02076895
PCSK1	1	0.59873321	0.006392688	190190.6433	0	0.020493181
PCSK7	2	0.7918578	0.0007383	2538.968733	0	0.020226317
PCTK1	3	0.9381329	0.011742691	641031.4498	5489.23408	0.02057023
PDAP1	2	0.82812889	0.002657602	33390.30716	0	0.020364339
PDCD4	2	0.71887653	0.000148594	106.3282907	0	0.020050508
PDCD6IP	8	1.24622779	0.01742395	1420523.354	5965.637378	0.020664609
PDE5A	1	0.59740928	0.003989151	74000.41531	0	0.020423302

PDGFB	8	1.24557174	0.004344085	89455.49588	3179.302787	0.020444338
PDGFD	1	0.58759653	0.002462446	28625.89597	0	0.020337408
PDGFRA	10	1.3161616	0.025216686	2974310.573	5832.144129	0.020642618
PDGFRB	23	1.42119047	0.054812806	14067788.36	13254.72889	0.020747998
PDIA3	5	1.08318224	0.007352623	251470.5118	2791.653707	0.020507953
PDIA6	1	0.52548049	0.000131141	82.12330847	0	0.020010785
PDLIM5	2	0.84263556	0.002164149	21819.84473	473.5442177	0.020413288
PDPK1	9	1.25704736	0.017143214	1370876.967	4742.85546	0.020636878
PDZK1	6	1.0718977	0.005534084	142357.0617	4383.462463	0.020493427
PEBP1	4	1.03216505	0.004246935	83906.78117	4438.908347	0.020466399
PELI1	1	0.57231784	0.001161799	6281.52743	0	0.020251993
PER1	4	0.86965555	0.004446941	91953.30863	4818.147981	0.020406699
PER3	2	0.61179169	0.000222262	233.1145398	0	0.020009612
PEX12	1	0.47466048	0.000135744	88.22126866	0	0.019969116
PEX5	4	0.90207404	0.003021594	42598.93494	4264.952529	0.020364825
PFKFB3	1	0.56393665	0.000361224	610.3811549	0	0.020115958
PFKP	1	0.58829399	0.000887419	3676.462767	0	0.020317317
PGF	3	0.91718974	0.001466562	10057.08859	0	0.020298231
PGK1	2	0.8328563	0.005050352	118546.3866	56.33830996	0.020474745
PGM1	1	0.4867531	3.93005E-05	9.110018289	0	0.019896264
PGR	11	1.32170088	0.031161203	4512824.35	3482.863836	0.020669863
PHB	9	1.30034395	0.04136558	7961905.883	2420.160949	0.020683137
PHB2	13	1.33901177	0.038566691	6912325.12	17566.13518	0.020685894
PHF1	2	0.77945968	0.000962175	4306.950205	193.7405638	0.020269548
PHF15	1	0.57063118	0.001248816	7264.38632	0	0.020287375
PHLDA1	4	0.98399514	0.004264769	84563.41069	4357.235123	0.020460266
PIAS4	12	1.34572252	0.043224752	8688212.409	18004.85918	0.020775269
PICALM	3	0.99136766	0.006920481	222725.0934	249.3178305	0.0204745
PIGP	2	0.3116381	6.25666E-06	2.414314065	3322	0.019627683
PIGQ	1	0.23769552	2.81078E-07	1.591046377	0	0.019259844
PIK3CD	3	0.72472891	0.004334741	88656.36516	5756.058957	0.020443359
PIK3R1	51	1.48045495	0.096045545	43179854	76149.87272	0.020855223
PIP5K1A	2	0.81597663	0.00415558	80303.66903	148.0491211	0.020429168
PIR	1	0.57945584	0.002740995	34926.19759	0	0.020322638
PITPNA	3	0.91885615	0.007587671	269444.8281	760.9010789	0.020553388
PKIB	4	0.98636606	0.0057828	155645.9455	3817.470558	0.020483836
PKMYT1	3	0.99450086	0.005159479	123843.6631	375.4289175	0.020481378
PLA2G4A	5	1.15743191	0.012342778	708696.8604	998.9223001	0.020596539
PLAC8	1	0.54122797	6.47828E-05	21.64178001	0	0.019954876
PLAGL1	3	1.00706378	0.023232416	2510686.15	0	0.020647362
PLAT	8	1.13115012	0.000800703	3127.639692	9559.075968	0.020311515
PLAU	11	1.2752124	0.001823287	15578.95181	13887.44979	0.020437974
PLAUR	8	1.18122566	0.005106817	122204.6956	11055.30212	0.020482607
PLEK	2	0.73174765	0.000950192	4203.146394	146.3844988	0.020276531
PLEKHF2	4	0.89628166	0.0007881	2893.257805	3587.078142	0.020228474
PLK1	29	1.39296957	0.028742312	3840456.861	60645.48655	0.020733381
PLSCR1	18	1.36683021	0.023390241	2554975.285	36372.61744	0.020712249
PLSCR4	2	0.81596505	0.001115581	5844.146728	314.36962	0.020316108
PLXDC1	1	0.55084184	0.000120549	70.93035494	0	0.020062055

PLXNB1	3	0.95012257	0.007441906	257983.2794	1209.415199	0.02052176
PLXNB3	2	0.32182638	2.76939E-06	2.319050581	6640	0.019495649
PMAIP1	5	1.11089506	0.005960158	165213.7289	1249.969768	0.020502781
PML	26	1.43808803	0.103063879	49379118.83	23706.99593	0.020843509
PMP22	1	0.53615303	9.83059E-05	47.13712603	0	0.01998361
PNRC1	1	0.59936649	0.009529691	422045.606	0	0.020541763
POLA2	10	1.25279925	0.005583672	144927.8248	18047.732	0.020545225
POLB	2	0.84347142	0.004838379	109075.5387	69.50910189	0.020412556
POLD1	3	0.97702664	0.008253129	317212.4802	682.3867462	0.020472535
POLD4	2	0.74162165	0.003758409	65851.065	0	0.020357782
POLDIP2	2	0.80708609	0.004103861	78463.44633	76.30831017	0.020372116
POLE2	2	0.81963451	0.001037089	5012.584707	474.6003356	0.02027918
POLQ	1	0.35594078	1.32896E-05	2.432642324	0	0.019762205
POLR2C	8	1.20246096	0.002073207	20009.41438	8952.487998	0.020403285
POLR2E	4	0.98923593	0.000416005	811.9101402	249.3126043	0.020144683
POLR2F	3	0.92723041	0.002461295	28175.98092	825.116919	0.020366526
POLR3C	1	0.36206411	1.1201E-05	2.25717597	0	0.019752372
POLR3H	3	0.56698045	0.000249329	293.9266952	13270	0.020139454
POR	2	0.59255428	0.004156189	80312.52178	3322	0.020446297
PPA1	2	0.84433227	0.012055333	676561.9306	0	0.020569487
PPARA	10	1.29463805	0.022417146	2335826.095	1373.477379	0.020595297
PPARD	7	1.23967402	0.032061074	4777115.486	2344.348179	0.020665609
PPFIA1	3	0.98530719	0.005291098	130257.0025	292.1180329	0.020451931
PPIL5	1	0.58210833	0.00199128	18450.57455	0	0.020303784
PPL	5	1.16118686	0.010112395	475579.376	3491.299801	0.020643117
PPM1G	3	0.93195039	0.001141582	6075.623561	1542.792431	0.020346377
PPP1CA	24	1.40829532	0.050025433	11637046.14	36672.74432	0.020764908
PPP1R15A	5	1.14997379	0.010138147	477989.9181	625.5276798	0.020551409
PPP2CB	11	1.25045455	0.003945287	72609.44982	4580.297098	0.020424035
PPP2R5C	2	0.60082325	0.009627718	430939.0659	3322	0.020521267
PQBP1	2	0.8308763	0.001207197	6796.940965	258.8688565	0.020356811
PRC1	2	0.85123649	0.005863002	159975.4376	196.7891202	0.020463455
PRCP	1	0.33987902	2.58993E-05	4.835889583	0	0.01982187
PRDX3	1	0.58157088	0.001673898	13046.38787	0	0.020332321
PREP	1	0.33987902	2.58993E-05	4.835889583	0	0.01982187
PRIM1	2	0.84532082	0.011393819	604424.1228	1389.564177	0.020568
PRIM2	1	0.56543911	0.000248321	289.9871905	0	0.020137554
PRKACB	1	0.49700925	0.00025979	316.5644185	0	0.020083534
PRKDC	25	1.43780789	0.08473917	33381680.75	15059.13269	0.020813012
PRLR	6	1.16484283	0.008904653	371828.8802	1085.619474	0.020502042
PRMT1	15	1.35953972	0.04407982	9030150.335	25600.2074	0.020740687
PRNP	8	1.21481344	0.004219931	83142.78195	7319.450931	0.020482361
PRPF3	1	0.57575687	0.00025011	294.5851227	0	0.020126157
PRPF31	1	0.58241143	0.0014783	10170.02802	0	0.020282554
PRPF6	5	1.15449298	0.022306301	2312335.139	4808.578923	0.020631391
PSAP	2	0.60034088	0.009352778	406808.3732	3322	0.020499087
PSEN1	16	1.36461275	0.028754577	3851155.696	14078.94261	0.020715768
PSMA4	4	1.04882651	0.003231288	48561.4203	200.5461696	0.020445073
PSMA5	4	1.05035162	0.003532965	58043.89859	889.5167462	0.02045953

PSMA7	8	1.24260322	0.013762224	881416.6272	16131.78343	0.020657857
PSMB1	6	1.06126893	0.001918467	17144.06341	3618.087474	0.020369199
PSMB2	2	0.78785827	0.001372317	8770.37995	0	0.020323606
PSMB3	5	1.03362063	0.00180478	15176.59135	45.89537153	0.020354869
PSMB4	1	0.58271549	0.001291238	7761.157645	0	0.020323364
PSMB5	7	1.16304786	0.006386802	190913.9415	7307.121961	0.020518307
PSMB7	5	1.03563867	0.001753586	14326.46645	1310.915638	0.02036021
PSMB8	2	0.74306422	0.000534707	1334.187709	257.9651897	0.020193775
PSMD1	7	1.16322565	0.004329814	87186.34788	3494.923836	0.02045953
PSMD12	3	0.84010915	0.000145925	110.5014104	0	0.020016417
PSMD14	6	1.02591671	0.000682813	2207.865059	1221.053195	0.020252474
PSMD2	5	1.09211067	0.004198173	81929.48348	3214.062505	0.020483836
PSMD3	4	0.92876567	0.000153492	122.4621746	717.7547238	0.020037563
PSMD7	8	1.12109168	0.002046234	19516.37264	6859.308125	0.020413532
PSME1	4	1.0316248	0.002288232	24380.81503	2919.430478	0.020438953
PSME3	8	1.22988201	0.022868735	2433167.707	10504.50847	0.02066661
PTAFR	1	0.36501649	5.72709E-05	17.55389387	0	0.0198433
PTBP1	2	0.82666267	0.004182053	81330.58866	310.6435811	0.020439932
PTGDS	1	0.60093811	0.006706974	211028.0891	0	0.020533609
PTGS2	7	1.23470031	0.018764752	1637248.495	3017.591098	0.020657107
PTHLH	4	1.06817368	0.007595592	268401.4773	1002.770705	0.02052324
PTP4A2	3	0.48842064	4.70251E-05	13.73538904	6642	0.019931344
PTPLB	1	0.55179248	0.000423771	840.1511785	0	0.020178498
PTPN13	7	1.19725295	0.011145901	578523.5631	5923.344389	0.020603003
PTPN2	10	1.32107472	0.039601291	7315598.587	3103.18616	0.020733381
PTPN22	2	0.84373424	0.004261158	85239.91251	6.716866727	0.020415974
PTPN6	24	1.42765005	0.061174234	17527644.79	20180.80844	0.020783111
PTPN7	1	0.59233201	0.00300818	42118.35613	0	0.02040109
PTPRC	16	1.37106864	0.018509756	1615018.297	21209.98816	0.020610715
PTPRCAP	2	0.84092258	0.001361149	8709.766017	663.1050734	0.020353655
PTPRF	9	1.28808287	0.017721083	1465175.953	4587.569587	0.020642868
PTPRN2	1	0.44913888	8.21276E-05	33.26319351	0	0.019899976
PTPRZ1	1	0.59873321	0.006392688	190190.6433	0	0.020493181
PTRF	3	0.5038616	0.00123166	7056.986057	6642	0.020272196
PTTG1	7	1.23463859	0.020962754	2043413.264	5233.088553	0.02066686
PUF60	10	1.26071171	0.002823262	37102.02587	9629.478299	0.020485065
PURA	3	0.99181421	0.011663148	632929.7606	62.05664137	0.020520773
PVR	1	0.21012527	5.50908E-08	1.590654638	0	0.019051339
PVRL2	1	0.56316415	0.000142923	97.69744325	0	0.020020407
Parp1	1	0.59011493	0.003029192	42664.23	0	0.020359481
Pebp1	1	0.58157088	0.001673898	13046.38787	0	0.020332321
Per1	2	0.80910586	0.004414471	90610.86928	78.49867772	0.020405968
Pfkp	1	0.58829399	0.000887419	3676.462767	0	0.020317317
Plek	1	0.57083629	0.000841223	3295.020352	0	0.020255838
Polq	1	0.59163149	0.002746897	35234.63826	0	0.020304991
Ppara	3	0.97179485	0.004317964	86684.37399	19.10813756	0.020326752
Ppard	1	0.57203265	0.001453095	9818.327607	0	0.020265698
QARS	1	0.33248187	3.94404E-06	1.67308375	0	0.019543638
RAB10	2	0.73917044	0.001594693	11854.25821	942.1767603	0.020280144

RAB17	4	0.94168359	0.001530448	10894.93603	4534.471222	0.020370415
RAB22A	1	0.50276592	6.75713E-05	23.15492074	0	0.01997753
RAB26	1	0.42801016	0.000129927	80.309377	0	0.020027219
RAB27A	4	0.98036887	0.003687485	63239.32831	3336.597003	0.020442135
RAB27B	2	0.63553591	0.000119548	69.22416896	34.74812084	0.019986418
RAB2A	1	0.55595671	0.000926618	3999.808002	0	0.020304991
RAB3D	2	0.81875019	0.005920452	162967.7577	0	0.020466399
RAB5B	1	0.21345389	8.11147E-08	1.592258442	0	0.019183224
RABAC1	6	1.00930109	0.001504104	10528.37308	9097.096828	0.020373575
RABEP1	2	0.81670715	0.004083984	77563.33236	948.9175847	0.020438708
RAC2	5	1.12481393	0.003727282	64657.8988	1150.62041	0.020458795
RAD21	3	0.52904193	0.000276668	359.1889357	6642	0.020086134
RAD23A	4	1.05751139	0.011748729	641618.048	1615.97756	0.020568495
RAD23B	2	0.81706052	0.003603561	60358.2644	50.6100656	0.020406943
RAD50	11	1.32138705	0.034514444	5544637.383	10332.97298	0.020617685
RAD51	16	1.36471679	0.044125636	9056718.161	16407.01687	0.020733633
RAD51AP1	1	0.57771232	0.001982329	18304.7367	0	0.020323606
RAD54B	4	0.98623388	0.006321279	185821.7389	4699.791308	0.020491951
RAD54L	1	0.57771232	0.001982329	18304.7367	0	0.020323606
RAD9A	9	1.2867663	0.028588451	3801792.318	5079.965972	0.020659357
RAE1	14	1.29815432	0.005527495	142156.8888	30731.38114	0.020540033
RALA	5	1.00959864	0.001633313	12418.53453	4676.209085	0.020365068
RALB	1	0.35217496	2.34081E-05	4.217350387	0	0.019831773
RALGPS1	2	0.79029716	0.000840892	3368.856835	175.1364306	0.020219129
RALGPS2	1	0.59087769	0.00238292	26408.51754	0	0.020401578
RALY	2	0.65407138	0.000248866	298.3821043	53.20101484	0.020085425
RANBP1	1	0.58536296	0.001499351	10459.37233	0	0.020350743
RAP1GAP	1	1.11023275	2.37285E-17	1.543080635	0	0.000585823
RAP2B	1	0.50283974	7.3376E-05	26.89692	0	0.01996935
RARA	19	1.40383926	0.055551713	14341794.56	6116.754268	0.020708731
RB1	54	1.48342828	0.142728223	94697676.78	70188.86608	0.020858281
RB1CC1	1	0.36858016	5.81257E-05	17.41846181	0	0.019929715
RBL1	17	1.3922042	0.06034286	16940414.14	2038.068013	0.020693167
RBM4	1	0.59624481	0.004571764	97293.52528	0	0.020423058
RBPMS	4	0.99564057	0.000984047	4507.688668	840.482645	0.020301369
RBX1	14	1.36083827	0.021517887	2152527.491	10245.48804	0.020662357
REC8	1	0.53466255	0.000420979	827.7664535	0	0.020133041
RECK	1	0.56775676	0.000140475	103.6566357	0	0.020021112
RELB	11	1.30933681	0.022772488	2411545.872	3967.564446	0.020549182
REPS2	2	0.54309893	0.000521052	1280.083265	3322	0.020222004
RET	9	1.24566014	0.025550239	3054172.627	7216.413525	0.020631889
RFC3	2	0.79994384	0.004065921	77062.64004	0	0.020357782
RFC4	4	1.01983981	0.010603472	523464.8077	363.5115081	0.020513375
RFC5	3	0.96311374	0.004595526	98393.45727	975.3997507	0.020431613
RFX5	1	0.59365601	0.004365683	88702.60588	0	0.020392803
RGS1	1	0.45148997	5.07774E-05	13.70307227	0	0.019920179
RGS4	2	0.57425401	0.001274822	7721.691592	3322	0.020233989
RHOB	1	0.48784785	4.68353E-05	12.24046996	0	0.019930413
RHOC	1	0.57804629	0.000627019	1853.511253	0	0.020200227

RHOD	1	0.57083629	0.000841223	3295.020352	0	0.020255838
RHOQ	1	0.26611666	5.05242E-07	1.643046556	0	0.019380769
RING1	7	1.08925937	0.010928117	555675.6842	7906.81394	0.020506475
RIOK3	1	0.5153362	8.61864E-05	36.87494188	0	0.019973322
RIT1	2	0.84483244	0.008496078	335485.1476	29.50164681	0.020540033
RMND5B	1	0.5187248	2.70452E-05	5.427698551	0	0.01978901
RND1	3	0.87130709	0.00136302	8817.226681	107.5292826	0.020244067
RNF103	3	0.8551752	0.000681938	2173.426632	1935.657178	0.020250792
RNF130	2	0.82405806	0.001264034	7431.17078	294.5766754	0.020310065
RNF139	1	0.58210833	0.00199128	18450.57455	0	0.020303784
RNF19B	1	0.51433642	0.000499358	1163.034873	0	0.020126632
RNF43	1	0.56366717	0.000652057	1984.803183	0	0.020236148
RNH1	2	0.54762368	0.000313059	459.6887288	6640	0.020155387
RNPS1	17	1.35385478	0.005567314	144207.6056	23204.37337	0.020528177
RPA1	16	1.38256111	0.037154663	6428732.905	7107.752819	0.020667361
RPIA	1	0.52175695	0.000157802	117.8397699	0	0.020036622
RPL10	2	0.85724441	0.008551322	342520.6211	0	0.020536079
RPL13A	1	0.44687465	0.00011665	65.25714786	0	0.019985014
RPL14	1	0.49640657	0.000191593	172.5596635	0	0.020060876
RPL18	1	0.58241143	0.0014783	10170.02802	0	0.020282554
RPL18A	1	0.59603049	0.006371527	188713.1298	0	0.0204128
RPL22	1	0.54932159	0.00053874	1352.701532	0	0.020192581
RPL24	1	0.56992061	0.000684994	2187.445381	0	0.020224639
RPL31	3	0.94161023	0.011780465	645159.0159	1040.659628	0.020572462
RPL34	1	0.56781426	0.000216715	221.8452422	0	0.020137791
RPN1	5	1.06434124	0.002271532	24224.50073	705.7394164	0.020322396
RPS27A	3	0.98167764	0.002467989	28334.01056	1327.05378	0.020408407
RPS6KA4	3	0.99104313	0.00683885	217462.2291	104.8077553	0.020470817
RPS6KA5	8	1.26769354	0.037103179	6398010.296	1632.676359	0.020657107
RPS6KB2	4	0.991852	0.009340326	408652.7156	3359.156882	0.020543247
RRM1	1	0.46292251	0.000623332	1810.162241	0	0.020220087
RRM2	3	0.86060286	0.013875045	895222.314	3417.098958	0.020625906
RSF1	1	0.59264131	0.004082311	77469.16377	0	0.020372846
RSRC1	1	0.58043325	0.000529605	1309.398852	0	0.020228953
RSU1	2	0.84278908	0.00226743	24026.02857	511.7787862	0.020431124
RTN2	1	0.5151867	0.000176574	147.216295	0	0.020030509
RUNX1	20	1.38534774	0.051625183	12389173.63	18919.46193	0.020676623
RUNX1T1	13	1.33937238	0.023251441	2514595.214	5840.564417	0.020612955
RUNX3	6	1.20619569	0.030300212	4268786.334	1002.804537	0.020631391
RUVBL1	15	1.33799997	0.016606304	1281949.619	22969.21978	0.020671866
RXRA	28	1.43601292	0.067428293	21130601.43	42015.99165	0.020770972
Rab17	1	0.57083629	0.000841223	3295.020352	0	0.020255838
Rara	1	0.57338218	0.002146196	21417.46655	0	0.020261609
Rb1cc1	1	0.46165841	0.000408537	778.4637299	0	0.020142544
Rbx1	3	0.96959043	0.001785338	14854.25079	90.50249545	0.02028858
Rhod	1	0.57083629	0.000841223	3295.020352	0	0.020255838
Rps27a	1	0.57083629	0.000841223	3295.020352	0	0.020255838
S100A1	5	0.94677508	0.000874809	3601.433474	3529.600054	0.020289063
S100A10	3	0.76479933	0.000575833	1545.792724	3507.870554	0.020226556

S100A6	1	0.55595671	0.000926618	3999.808002	0	0.020304991
S100A8	7	1.18716	0.013768952	882062.5863	8830.006277	0.020630144
S100A9	4	0.98255131	0.007935589	294727.906	4475.913998	0.020585114
S100P	2	0.78930795	0.001650739	12863.25979	511.2654191	0.020370658
SAFB	5	1.11771954	0.010877265	549899.0121	4478.237401	0.020574941
SALL2	1	0.47875113	3.21431E-05	6.597342383	0	0.019854609
SAMD4B	1	0.59087769	0.00238292	26408.51754	0	0.020401578
SAP18	9	1.1841113	0.011296346	594101.0243	10468.20338	0.020488753
SARS	1	0.59603049	0.006371527	188713.1298	0	0.0204128
SART1	2	0.75559015	0.000277064	361.0320332	704.2000729	0.020153959
SAT1	9	1.26757885	0.020284691	1913613.673	11612.25834	0.020650609
SATB1	6	1.20136715	0.027668096	3560517.143	895.456644	0.02060549
SAV1	1	0.49731248	0.000698957	2277.029118	0	0.020207401
SCAMP1	5	1.04148361	0.01845121	1582518.958	8724.106711	0.020700193
SCARB1	3	0.88483617	0.000444792	924.8611394	554.4165925	0.020223202
SCARB2	1	0.57695036	0.000117838	78.28912698	0	0.020026984
SCGB1D2	1	1.11023275	-1.04796E-17	1.543080635	0	0.000585823
SCGB2A2	1	1.11023275	3.53368E-17	1.543080635	0	0.000585823
SCNN1A	4	0.96125725	0.004499316	94115.67648	3895.542305	0.020449971
SCP2	2	0.78356652	0.002874713	38769.80738	37.77354726	0.020368713
SDC1	4	1.00770733	0.000213039	224.5498775	328.1939009	0.020109323
SDC2	7	1.20227368	0.00285652	38634.42914	5158.248142	0.020478184
SDC4	3	0.967211	0.002391274	26680.7834	597.5196298	0.02036847
SEC14L1	1	0.57188237	0.001077074	5400.80811	0	0.020310306
SEC16A	1	0.56045328	0.000649071	1962.406734	0	0.020150866
SEC23IP	2	0.74162165	0.003758409	65851.065	0	0.020357782
SELENBP1	4	1.03969219	0.011447999	609156.4688	3060.649403	0.020568
SELL	3	0.9824986	0.002249353	23933.64248	637.9514537	0.020388176
SEMA3B	1	0.51224502	0.00010322	52.14942235	0	0.019959776
SEMA3C	1	0.51224502	0.00010322	52.14942235	0	0.019959776
SEMA3F	3	0.77028141	0.000456558	977.5271295	3322	0.020177305
SEPP1	1	0.60093811	0.006706974	211028.0891	0	0.020533609
40788	3	0.79301572	0.00072689	2464.39833	3394.977393	0.020249351
40792	1	0.44263032	3.26553E-05	6.644920653	0	0.019858074
SERINC3	4	0.90461848	0.001844579	15820.52024	4019.222144	0.020323606
SERPINA3	4	0.91013561	0.003194556	48011.09057	5559.097467	0.020412312
SERPINA5	3	0.7895067	0.00011812	73.15329534	3322	0.020067479
SERPINB9	5	1.1396653	0.016830542	1317735.157	4571.828454	0.020647112
SERPINE1	5	1.10999467	0.000356392	619.3538877	672.9078428	0.020212905
SERPINE2	2	0.78444163	0.000134942	91.74188615	62.02310159	0.020067243
SERPINH1	2	0.83299347	0.011284652	592776.2726	3181.303893	0.020575437
SERPINI1	1	0.53127382	3.59713E-05	9.015894258	0	0.019917855
SF3B2	2	0.81802049	0.007738142	278385.9544	237.1400122	0.020520773
SFI1	2	0.80985475	0.000269759	342.9110437	370.3712313	0.020153959
SFRP1	1	1.11023275	-1.07435E-18	1.543080635	0	0.000585823
SFRS11	3	0.96966512	0.001869756	16272.33228	183.2106063	0.020378926
SFRS4	3	0.88415328	0.000166125	134.3289864	78.29297919	0.020016182
SFRS5	1	0.5356503	0.000129412	80.13180023	0	0.019987354
SFRS6	5	0.99605896	0.000504744	1196.941912	4062.932491	0.020177066

SGK3	1	0.55749826	0.000770153	2780.515696	0	0.020230632
SH2B1	4	1.06511997	0.00461668	100458.9851	359.575348	0.020421347
SH3D19	4	1.02820846	0.001075278	5472.985893	711.7269809	0.0202568
SHC1	46	1.47359382	0.083041096	32350907.54	45832.88693	0.020811997
SHCBP1	1	0.59636493	0.003730592	65960.5962	0	0.020398896
SIAH2	2	0.85470404	0.006534171	198575.5825	133.8430963	0.020506475
SIGIRR	3	0.92956164	0.001470349	10084.16426	162.340808	0.020295576
SIGMAR1	1	0.34998566	1.33248E-05	2.435226424	0	0.019771819
SIN3A	24	1.42371144	0.059273201	16346491.14	12919.48603	0.02070848
SIP1	5	1.00852675	0.000312479	464.9398695	1421.161397	0.020170867
SKAP1	3	0.99847239	0.009382867	412933.2778	96.44975635	0.020545719
SKIL	23	1.39359762	0.027408214	3491580.619	46562.26952	0.020711244
SKP1	14	1.29881624	0.014745052	1010489.92	19521.3162	0.020617934
SLC16A1	3	0.840872	0.000580771	1593.181975	310.3887421	0.02021889
SLC16A4	1	0.51207105	0.000195424	182.3245412	0	0.020074795
SLC1A1	1	1.11023275	1.7536E-17	1.543080635	0	0.000585823
SLC22A5	2	0.78452191	0.001292799	7837.988245	0	0.020316591
SLC25A20	1	0.37232554	0.000186715	163.8405245	0	0.020047447
SLC2A1	5	1.09110341	0.005665262	149178.3354	2067.275115	0.020500072
SLC3A2	5	0.96607709	0.007226885	245199.1675	11561.01183	0.020552646
SLC6A8	1	0.48858934	0.000283276	379.9784752	0	0.020098905
SLC7A7	1	0.49180489	0.000324665	502.2039913	0	0.020149677
SLC9A3R1	12	1.3179685	0.023242972	2525465.537	10117.40103	0.020687649
SLC9A7	1	0.51062579	0.000828914	3197.332434	0	0.020291474
SLIT2	1	0.43961348	1.77057E-05	3.139692584	0	0.019810598
SLPI	5	1.06334677	0.00128366	7733.779903	6763.33092	0.020369199
SMAD3	67	1.49071101	0.165984836	128052390.4	140013.0098	0.020935794
SMARCA2	18	1.39220144	0.055612716	14379766.77	7196.519515	0.020688903
SMARCA4	31	1.45204585	0.104213761	50489391.24	31591.42424	0.020835876
SMARCC1	13	1.35970709	0.053708591	13413784.66	2745.776591	0.020714511
SMARCC2	10	1.29080992	0.024195026	2721957.747	7353.413291	0.020639373
SMARCD2	2	0.86369789	0.0204794	1951474.272	0	0.020604246
SMARCE1	12	1.325949	0.034090422	5401162.001	6501.259093	0.020683638
SMC2	2	1.1455403	7.41239E-17	2.70827166	0	0.000586166
SMC3	6	1.14662328	0.00937077	408570.1336	6775.494594	0.020535338
SMC4	2	1.1455403	-2.75514E-17	2.70827166	0	0.000586166
SMS	1	0.56139909	0.000917967	3919.800974	0	0.020246949
SNRNP200	3	0.95460128	0.000639616	1909.437867	332.2655226	0.020231351
SNRPA1	2	0.48842823	5.51525E-05	16.62384208	3322	0.01991832
SNRPB2	1	0.32834293	2.47771E-06	1.624090278	0	0.019539611
SNRPF	2	0.7371012	0.000124095	75.61256799	0	0.020040386
SNRPG	3	0.87614368	0.000354388	589.2535175	606.2608467	0.020155863
SNRPN	1	0.60238036	0.011145498	578362.3686	0	0.020555863
SNX1	5	1.12127898	0.011377518	607604.7736	3003.614613	0.020577421
SNX3	1	0.49055273	0.00020213	191.8640377	0	0.020050979
SOCS3	13	1.34979726	0.022362955	2350502.735	6707.329184	0.020608724
SOCS5	2	0.85016387	0.007981901	299427.2595	64.20420909	0.020538056
SOD1	3	0.78961287	0.003013679	42238.30742	3439.586258	0.020416951
SOD2	1	0.5793091	0.001583791	11691.85093	0	0.020275326

SORBS1	4	1.06923841	0.002892215	38999.1185	614.7930987	0.02038915
SORL1	2	0.77521866	0.000114282	64.18127588	208.5261227	0.02008448
SOX10	4	1.07023519	0.012484145	724485.8928	310.9043222	0.020502288
SOX17	1	0.59873321	0.006392688	190190.6433	0	0.020493181
SOX4	1	0.59549088	0.004218019	82724.09062	0	0.020428435
SOX9	4	1.08840281	0.020941785	2039070.201	189.1110445	0.020579406
SPAG5	2	0.74280145	0.00257137	30772.10918	42.20487273	0.020372603
SPARC	14	1.34020452	0.002258667	24848.84869	5813.658845	0.020416462
SPARCL1	3	0.98729771	0.008127857	309007.1617	1198.658746	0.020581391
SPC25	1	0.55213724	0.000441765	910.7777838	0	0.020118803
SPDEF	1	0.59936649	0.009529691	422045.606	0	0.020541763
SPG7	3	0.89170862	0.001204901	6807.693352	1186.041303	0.020319977
SPI1	10	1.30366759	0.039893552	7399359.435	2124.831308	0.020638375
SPOCK1	3	0.95901704	0.000325264	527.0260833	68.3895921	0.020154911
SPOCK2	2	0.47360667	6.15205E-05	20.36938534	9954	0.019871713
SPOP	3	0.97257738	0.004027224	75408.18028	455.4809665	0.020420859
SPRED2	1	0.57624561	0.001274927	7740.297697	0	0.020244547
SPRY1	1	0.54712754	7.02044E-05	25.31000427	0	0.020021816
SPRY2	1	0.57809071	0.001050798	5186.761959	0	0.020303059
SRC	62	1.4899825	0.135696721	85827202.45	124610.9933	0.020901956
SRCAP	2	0.85377158	0.012131452	684231.7014	0	0.020488999
SREBF1	6	1.2007487	0.025792377	3091928.772	771.6452207	0.020648361
SRGN	1	0.58674723	0.001241242	7316.392548	0	0.020310306
SRPK1	8	1.15117579	0.002880647	38623.82667	8004.949517	0.020383793
SS18L1	2	0.8619794	0.018076299	1519178.078	0	0.020554378
SSH3	1	0.59260624	0.001415799	9332.977794	0	0.020359724
SSR1	2	0.73506764	0.000564259	1486.684269	186.703977	0.020244788
ST13	1	0.46101984	0.000536517	1343.510384	0	0.020182553
STAG2	1	0.34620981	1.24292E-05	2.368346619	0	0.019701079
STAMBPL1	4	0.95204269	0.00122519	6996.435027	7155.025703	0.020311515
STARD13	2	0.76470455	0.000699095	2289.983819	23.81863692	0.020259444
STAT1	41	1.47075078	0.102260851	48634078.94	69598.23088	0.020870012
STAT3	47	1.47920089	0.144605336	97218628.96	61136.13467	0.020870777
STAU1	7	1.20193638	0.006563499	200268.9426	3943.322676	0.020514854
STC2	3	0.77649642	0.000284504	380.3022348	3475.628029	0.020166578
STK24	15	1.32912513	0.01235592	709991.3032	8829.36	0.020599522
STK4	4	0.98756106	0.015558436	1125939.99	3747.578205	0.020612706
STMN1	3	0.8562442	0.009093823	384553.8377	3369.971038	0.020545225
STRA13	2	0.8614725	0.015363517	1098336.514	0	0.020605987
STRAP	5	1.14417736	0.010093757	473517.6118	2739.920792	0.020608475
STRN	12	1.29005329	0.015056731	1054350.953	10416.31692	0.020627152
STRN3	10	1.23227419	0.003028985	42891.03759	851.9067329	0.020400359
STT3A	2	1.1455403	1.38379E-17	2.178183557	2	0.000586166
STUB1	13	1.35369929	0.0642276	19171684.74	6120.835301	0.02080667
STXBP1	2	0.84430408	0.002676198	33393.43844	83.91273937	0.02043357
STXBP2	3	0.83836048	8.82049E-05	42.66238355	34.43319257	0.020022991
STXBP3	3	0.83836048	8.82049E-05	42.66238355	34.43319257	0.020022991
SUB1	4	1.09802633	0.024577367	2808866.436	119.7550649	0.020611461
SUZ12	3	0.99082295	0.007452138	258234.3995	434.1458263	0.020530152

SYNCRIP	5	1.10453198	0.003286033	50202.58606	1428.610439	0.020454872
SYNJ2BP	3	0.94392321	0.001464509	9990.364751	1893.377668	0.02039256
SYNRG	2	0.76112479	0.000923747	3972.676858	105.4495383	0.0203178
SYTL2	3	0.84065253	0.002418405	27224.17533	3197.937963	0.020381846
Safb	1	0.49945828	2.26754E-05	4.293191823	0	0.019788551
Sh2b1	1	0.56655542	0.000611977	1747.029436	0	0.020184463
Shc1	1	0.57385792	0.001272249	7689.162387	0	0.020233509
Sin3a	3	0.91586845	0.002293048	24497.84757	5.434059764	0.020252714
Sip1	1	0.59914918	0.00745681	258503.9824	0	0.020517813
Skil	1	0.5125358	0.000173474	142.4672321	0	0.020101508
Slc7a7	1	0.49180489	0.000324665	502.2039913	0	0.020149677
Smad3	1	0.59914918	0.00745681	258503.9824	0	0.020517813
Smarca2	1	0.59603049	0.006371527	188713.1298	0	0.0204128
Smarca4	1	0.59796927	0.006412015	191202.5703	0	0.020443359
Smarce1	1	0.5848568	0.003663441	62388.94308	0	0.020353655
Stat1	1	0.57083629	0.000841223	3295.020352	0	0.020255838
Stat3	1	0.60038967	0.009608287	429199.4929	0	0.020520773
TACC1	7	1.18643308	0.005356488	133562.7161	3867.514109	0.020424768
TACC3	2	0.83683304	0.003293096	50455.22476	102.7688356	0.020380143
TAF15	5	1.08364757	0.002682107	33443.61991	1957.409393	0.020385497
TAF2	2	0.64711136	0.000183065	158.8900815	0	0.01998478
TAF4	3	0.80895604	0.001818881	15386.56279	1514.540733	0.020294128
TAF7	4	0.91304275	0.002256044	23662.39697	2214.177212	0.020322638
TAGLN	1	0.49603885	0.000356504	601.2125041	0	0.020180883
TAOK3	1	0.59163149	0.002746897	35234.63826	0	0.020304991
TAP1	5	1.09121896	0.010704127	532612.9023	6423.064624	0.020575189
TBCB	1	0.58421517	0.001002374	4683.027446	0	0.020306199
TBL1X	3	0.9740522	0.00744749	258046.9536	191.882189	0.020515101
TBXA2R	1	0.59087769	0.00238292	26408.51754	0	0.020401578
TCEA1	5	1.09371933	0.006262316	182402.4621	1233.098033	0.020438708
TCEAL1	5	1.06071877	0.001735453	14029.16777	958.7951311	0.020357296
TCEAL4	2	0.79649593	0.001080339	5442.334831	0	0.020313448
TCEB1	9	1.21480448	0.006116618	173929.7938	5442.788914	0.020483836
TCEB2	5	1.07623104	0.001917919	17206.6228	846.3534594	0.020292922
TCF12	5	1.12518677	0.019373851	1745152.801	690.8878121	0.020559329
TCF4	11	1.31784315	0.041923464	8167963.288	6039.578816	0.020693417
TCF7L1	2	0.84224775	0.006884276	220597.4409	0	0.020512882
TCOF1	1	0.59469486	0.003589563	60056.60715	0	0.020357539
TCP1	11	1.28220217	0.007853123	286916.9996	4103.987019	0.020551409
TDG	13	1.36004891	0.072209019	24235965.95	6064.258737	0.020775016
TEAD4	3	0.70963697	0.000618729	1783.550671	3368.665256	0.020181599
TENC1	3	0.9559581	0.001723732	13970.66006	161.7557803	0.02032409
TERF1	10	1.28383765	0.012825373	766339.2396	8334.496406	0.02056032
TES	4	0.89537539	0.001366645	8766.301673	13327.01358	0.02026209
TFAP2A	8	1.27151213	0.040220679	7519463.165	2519.831512	0.020737663
TFAP2B	4	0.95989907	0.005435891	137402.3827	3819.612269	0.020457078
TFAP2C	3	0.98838166	0.016486263	1264672.074	106.3272883	0.020606485
TFDP1	9	1.27988552	0.029258594	3984587.165	1142.340174	0.020621172
TFF1	1	0.33524596	5.53319E-05	15.89109393	0	0.019880044

TFPI2	1	0.58282536	0.001231304	7053.65598	0	0.020302093
TFRC	2	0.2712497	1.80557E-06	2.298478368	3322	0.019548114
TGFB1	18	1.37065097	0.003778828	67566.63193	15374.7073	0.020503273
TGFB1I1	9	1.28815332	0.025869766	3110400.652	4124.949618	0.020672617
TGFB2	7	1.17379311	0.002350727	25929.51792	1187.955406	0.02043088
TGFB3	5	1.07232555	0.001755258	14393.04947	759.1299126	0.020370901
TGFBR2	20	1.4037522	0.032686719	4968907.626	36171.47121	0.020765413
TGFBR3	5	1.10370945	0.00195099	17815.19247	144.1304876	0.020396215
TGIF2	2	0.86121013	0.014081607	922156.9018	28.23866668	0.020575933
TH1L	9	1.20103874	0.00496545	114654.5383	10092.20325	0.020428679
THBD	1	0.44153497	5.30651E-06	1.856907789	0	0.019683133
THBS1	17	1.3604724	0.002623015	33526.27373	11127.1353	0.020425013
THBS2	2	0.83051917	0.000280862	405.4041882	54.50266057	0.020143257
THOC1	1	0.59796927	0.006412015	191202.5703	0	0.020443359
THOC4	1	0.36386508	8.07344E-05	32.01743107	0	0.019904384
THOP1	2	0.77345815	0.001337628	8320.883734	87.20365971	0.020339589
THY1	1	0.56174207	9.51666E-05	46.49537941	0	0.01999672
TIMELESS	6	1.08512855	0.006471681	195161.5466	2529.619663	0.020419149
TIMP1	3	0.95964468	0.00030207	459.877358	126.2413276	0.020138979
TIMP2	5	1.09443495	0.000939043	4156.536513	4185.021761	0.020357539
TIMP3	6	1.12112989	0.000580212	1655.06417	4074.503375	0.020226077
TIMP4	2	0.81898651	0.000271509	366.278539	0	0.020149914
TIPRL	1	0.55619773	0.000177241	151.7494334	0	0.020026044
TK1	5	1.12409255	0.017037851	1350726.678	2695.04334	0.020639124
TLE2	1	0.58137743	0.002319243	25018.55936	0	0.020268826
TLE3	1	0.56992061	0.000684994	2187.445381	0	0.020224639
TLR2	5	1.13926282	0.006958205	227048.1793	1068.243895	0.020508939
TM4SF1	3	0.92079216	0.002826385	37144.31963	166.2032045	0.020432347
TMBIM4	3	0.7773146	0.000321027	482.8061979	634.2871638	0.020135891
TMBIM6	1	0.59065493	0.002189443	22302.08792	0	0.020389637
TMEM33	1	0.5343439	4.29934E-05	10.73663905	0	0.019931576
TMEM49	1	0.58823865	0.001673396	13089.82011	0	0.020370658
TMPO	5	1.07890323	0.009154266	389725.0404	3802.167727	0.020536327
TNC	3	0.99345966	0.007863972	290648.8684	0	0.020567256
TNFAIP1	2	0.75230762	0.00089486	3768.934858	232.5076605	0.020267864
TNFAIP3	9	1.20443792	0.006954436	224978.6416	16513.7728	0.020557596
TNFAIP8	1	0.3283395	2.11259E-06	1.669998255	0	0.019552145
TNFRSF10A	9	1.26606175	0.005906782	162651.6508	4545.65416	0.020488507
TNFRSF10B	9	1.27324217	0.007893525	291936.3256	1105.014157	0.02052176
TNFRSF12A	2	0.75294586	0.000650765	1980.186423	0	0.020226077
TNFRSF1B	4	1.08265194	0.009057373	382125.7903	408.7787547	0.020585114
TNIP1	1	0.54690373	0.000312426	457.1150782	0	0.020154435
TNKS	4	1.00112351	0.00131458	8059.429981	616.8440971	0.020296058
TNKS2	2	0.7694819	0.000635232	1890.234774	0	0.020163481
TNNI3K	1	0.51480803	0.000453136	964.0749635	0	0.020180645
TNPO2	1	0.42051457	0.000194737	183.5205698	0	0.020044622
TNRC6B	1	0.45554368	0.00050488	1191.205907	0	0.020170629
TOB1	4	1.03601138	0.010984705	560910.7405	3515.13974	0.020592812
TOM1L1	5	1.08087722	0.007214399	243545.6805	4901.700374	0.02054844

TOMM20	3	0.9627937	0.003233165	48597.46789	838.6545867	0.0204745
TOP1	8	1.26784928	0.033229244	5134308.07	3478.161225	0.020690157
TOP2A	7	1.24709037	0.044382834	9162519.853	6491.365396	0.020677124
TOP3A	2	0.84300299	0.003690443	63466.42651	0	0.020371873
TOP3B	4	0.85457052	6.41881E-05	23.5323603	7219.63808	0.020007032
TOPBP1	7	1.23908184	0.020034599	1866793.983	817.8755297	0.020608226
TP53	125	1.51087504	0.248093175	286278191.5	347257.526	0.020975412
TP53BP2	6	1.19947811	0.016804186	1313991.289	4625.806251	0.020660357
TP53INP1	2	0.84794918	0.012938978	779410.1552	0	0.020561311
TPD52	3	0.60117796	0.000447595	938.1769676	0	0.020142544
TPD52L1	5	0.85844599	0.009068041	382351.8186	10139.12307	0.02054473
TPD52L2	3	0.60117796	0.000447595	938.1769676	0	0.020142544
TPI1	2	0.85749335	0.008765874	358477.623	315.6942415	0.020591819
TPM1	5	1.14131717	0.015953416	1184641.557	1699.202259	0.020668612
TPM2	3	0.97971094	0.008504028	336116.428	281.828682	0.020540033
TPM4	1	0.56168059	0.000411053	797.1012718	0	0.020154435
TPX2	2	0.73715447	0.00164287	12570.93646	490.227344	0.020329899
TRAF3	8	1.2182871	0.012799276	762966.0812	7899.616531	0.020631889
TRAF4	8	1.23513247	0.009696536	436999.6271	7379.595149	0.020595049
TRAF5	4	0.99086116	0.001686416	13237.78394	493.032592	0.020378683
TRAF7	1	0.51433642	0.000499358	1163.034873	0	0.020126632
TRAK1	2	0.58323393	0.001233794	7082.986677	3322	0.020302576
TRAK2	1	0.36862412	5.54278E-05	15.95252823	0	0.019909259
TRAM1	1	0.60017776	0.009919944	457388.8922	0	0.020548935
TRAP1	4	1.00124544	0.009573171	425918.5381	3799.581496	0.020568991
TRIM2	1	0.5187248	2.70452E-05	5.427698551	0	0.01978901
TRIM22	1	0.58421517	0.001002374	4683.027446	0	0.020306199
TRIM29	3	0.85391033	0.005465119	138847.5882	3751.271393	0.020499826
TRIP13	9	1.17724939	0.001442033	9691.735848	7011.693919	0.020350015
TRIP6	11	1.27851288	0.009149825	391329.2708	10730.92231	0.020557596
TROAP	2	0.36181511	2.72416E-05	5.763367585	6640	0.019784423
TROVE2	1	0.55821784	0.000126834	77.42051054	0	0.020084716
TSC22D3	5	1.13094803	0.013849061	891628.6281	3700.065619	0.020478921
TSNAX	2	1.1455403	-5.06407E-17	2.178183557	2	0.000586166
TSPAN4	4	1.01123808	0.000591471	1664.926398	347.5720276	0.020222962
TTC3	1	0.56971106	0.000167068	133.8829339	0	0.020147061
TTC8	1	0.44025285	1.17315E-05	2.360451843	0	0.019719286
TTF1	1	0.33524596	5.53319E-05	15.89109393	0	0.019880044
TTK	3	0.97787028	0.003851214	68946.9109	66.64253224	0.02042819
TUBA1A	4	1.00083501	0.008972603	376133.207	2442.837583	0.020589087
TUBB	17	1.39072986	0.038184071	6776367.733	23413.80245	0.020756828
TUBG1	4	1.08708244	0.015066789	1055326.781	3036.518315	0.020621172
TUSC3	1	0.58538039	0.002247375	23509.72524	0	0.020353655
TXNIP	4	0.99848579	0.011853198	653653.3811	3430.184195	0.020529411
TXNRD1	1	0.50006774	0.000532501	1322.902326	0	0.020127343
Tpd52	3	0.60117796	0.000447595	938.1769676	0	0.020142544
Tuba1a	3	0.93677715	0.001632959	12413.35549	524.6371654	0.020395971
UBE2A	3	1.00532894	0.017776162	1471262.787	8.76773671	0.020601262
UBE2B	1	1.11023275	-5.50911E-17	1.543080635	0	0.000585823

UBE2C	3	0.95109108	0.001784517	14853.12315	0	0.020245028
UBE2I	47	1.46826866	0.09389097	40974103.14	103003.6172	0.020842745
UBE2L3	6	1.05703756	0.011115457	574689.15	8623.297608	0.02052867
UBE2N	13	1.28885245	0.014514457	980406.7958	29609.25861	0.020642618
UBE2O	1	0.5125358	0.000173474	142.4672321	0	0.020101508
UBE2T	3	0.84918911	0.000298592	418.7816375	156.8538683	0.020106481
UBE2V2	3	0.97463156	0.007751045	281108.2493	2648.866894	0.020581639
UBE2W	8	1.07574083	0.000602013	1698.969561	7952.168767	0.020177543
UBE4B	2	0.77191541	0.004055427	76479.57739	622.7191374	0.020424768
UBL4A	2	0.81880942	0.001430527	9535.219091	208.76285	0.020357053
UBL5	4	0.85617617	0.000639811	1907.181667	7039.320096	0.02020214
UBR2	1	1.11023275	-2.86718E-17	1.543080635	0	0.000585823
UBR5	6	1.18468351	0.006944932	224231.3244	475.7571609	0.020485311
UCHL1	7	1.20520472	0.027075307	3406959.2	13226.95014	0.020744468
UGDH	1	1.11023275	-3.05304E-17	1.543080635	0	0.000585823
UHRF1	2	0.84073983	0.007884263	289336.4889	0	0.020477939
ULK1	1	0.27116129	1.80193E-06	1.658560872	0	0.019547667
UNC84B	1	0.58823865	0.001673396	13089.82011	0	0.020370658
UNG	1	0.59469486	0.003589563	60056.60715	0	0.020357539
USP1	7	1.16029643	0.003853597	69281.49918	10892.05641	0.020460021
USP36	6	0.92809873	0.002665749	33095.15757	10519.67084	0.020380873
USP42	7	0.98769374	0.00195027	17726.12077	14234.85688	0.020315866
USP7	25	1.40157014	0.022312351	2315316.624	43836.02871	0.020715517
UTP18	1	0.48176887	0.000119758	68.79610757	0	0.019984546
Ube2i	2	0.80058655	0.00204799	19521.29762	30.68838315	0.02032167
Ube2o	1	0.5125358	0.000173474	142.4672321	0	0.020101508
VAV1	27	1.43225509	0.051556379	12425095.01	25547.25855	0.020763645
VAV3	6	1.20677268	0.020634112	2006632.202	61.07715748	0.020592812
VCAM1	2	0.84290677	0.002173071	22320.36778	0	0.020373575
VCAN	11	1.25355907	0.001697995	13736.72519	8351.302477	0.020354141
VCL	9	1.28188099	0.018415026	1581310.006	3555.696089	0.020618681
VEGFA	11	1.26355608	0.007844599	286210.7682	22752.96813	0.020569239
VEZT	3	0.98919161	0.009764577	444106.6427	0	0.020530646
VGLL1	1	0.41538869	2.77962E-05	5.251877648	0	0.019792911
VIM	25	1.41717134	0.027514317	3518592.387	52098.07297	0.020740939
VPS37C	3	0.94789125	0.002633562	32490.36489	45.1232513	0.020414265
VPS37D	2	0.80453981	0.001850797	16015.8669	0	0.020382576
VPS72	1	0.56316415	0.000142923	97.69744325	0	0.020020407
VRK1	2	0.82742054	0.011537884	619711.1011	208.2257381	0.020561558
VTA1	7	1.08886366	0.003512584	57378.48879	9157.377286	0.020435038
VWF	3	0.97392999	0.001337359	8427.303634	672.0561443	0.020345892
Vim	1	0.58829399	0.000887419	3676.462767	0	0.020317317
WAPAL	1	0.34620981	1.24292E-05	2.368346619	0	0.019701079
WARS	2	0.69342988	0.000539942	1361.645673	732.0990442	0.020235188
WBP11	9	1.27795123	0.015082856	1061813.704	8935.23632	0.02064911
WDR33	5	1.15622984	0.017663026	1451689.958	2885.986824	0.0206279
WDR6	1	0.26611666	5.05242E-07	1.643046556	0	0.019380769
WDR61	2	0.82541259	0.00175919	14389.45743	440.2021254	0.020340558
WFS1	1	0.56781426	0.000216715	221.8452422	0	0.020137791

WHSC1	4	1.09815386	0.023182998	2499249.765	37.05345601	0.020590577
WIPF1	3	0.97579214	0.002442987	28337.93949	57.52067039	0.020365554
WNK4	1	0.33975713	3.00975E-05	6.056058059	0	0.019791534
WNT5A	1	1.11023275	-4.84162E-17	1.543080635	0	0.000585823
WRN	13	1.35452053	0.038118213	6766896.175	2563.716602	0.020673118
WSB2	2	0.79092548	0.001020015	4839.920156	148.3439166	0.020260647
WWP1	7	1.20630285	0.004011709	74813.69642	1953.345095	0.020470571
WWTR1	2	0.72776053	0.002347039	25622.09344	546.6361932	0.020269789
Wdr61	1	0.57083629	0.000841223	3295.020352	0	0.020255838
XBP1	2	0.85420987	0.012630691	741454.6088	18.4437115	0.020565026
XPNPEP1	1	0.55666113	0.000250844	294.8671997	0	0.020142544
XPO5	4	1.05046753	0.00182907	15586.3039	1436.958783	0.020387932
XRCC6	33	1.44801941	0.092327954	39620744.54	64246.40065	0.02086083
Xrcc6	1	0.52454293	0.000212779	212.4400783	0	0.020110745
YAP1	7	1.20726152	0.011397767	603956.2323	7063.343741	0.020584866
YBX1	8	1.26554129	0.030497032	4324806.956	7355.162588	0.020718534
YEATS4	5	1.10746292	0.001873244	16323.17973	1394.733358	0.020357053
YWHAZ	35	1.44053809	0.053042603	13077442.03	66910.21648	0.020814789
YY1	8	1.26113171	0.03388288	5339129.379	941.007003	0.020623913
ZBTB16	30	1.44321319	0.067631751	21261902.22	72137.4356	0.020800078
ZBTB43	1	0.57577454	0.000852463	3401.762953	0	0.020151104
ZEB1	3	0.96211659	0.017165801	1369877.076	1295.661791	0.020591322
ZEB2	1	0.59914918	0.00745681	258503.9824	0	0.020517813
ZER1	1	0.56393665	0.000361224	610.3811549	0	0.020115958
ZFAND3	1	0.44687465	0.00011665	65.25714786	0	0.019985014
ZFP106	1	0.58282536	0.001231304	7053.65598	0	0.020302093
ZMYND8	2	0.85048696	0.005684141	150286.0643	119.3701097	0.020470571
ZNF148	4	1.01126213	0.027305461	3465742.478	10036.81704	0.020679128
ZNF155	1	0.56992061	0.000684994	2187.445381	0	0.020224639
ZNF263	1	0.47305183	3.54051E-05	7.555070026	0	0.019837996
ZNF398	1	0.60017776	0.009919944	457388.8922	0	0.020548935
ZNF451	1	0.59549088	0.004218019	82724.09062	0	0.020428435
ZNF580	1	0.49697569	0.000283981	377.2099341	0	0.020091335
ZNF638	5	1.10062977	0.001518942	10793.23632	1882.392708	0.020374305
ZNF652	3	0.93598818	0.00201849	18995.39952	0	0.020222243
ZNF76	2	0.86171632	0.016233084	1225420.044	19.92920927	0.020563292
ZWILCH	1	1.11023275	-1.39268E-16	1.543080635	0	0.000585823
ZWINT	1	0.55213724	0.000441765	910.7777838	0	0.020118803
ZYG11B	2	0.80208626	0.000636011	1888.159734	0	0.020145634
cdc2	1	0.26962007	2.61657E-06	1.623838733	0	0.019551473
kpna2	1	0.54932159	0.00053874	1352.701532	0	0.020192581
mcm2	1	0.56798881	0.000714608	2398.354711	0	0.020145396
spi1	1	0.57498241	0.002166147	21835.14203	0	0.02032772
