# Supplementary figures and images for: Integration of breast cancer gene signatures based on graph centrality
Source: BMC Syst Biol. 2011 Dec 23;5(Suppl 3):S10. doi: 10.1186/1752-0509-5-S3-S10 (PMC3287565; doi:10.1186/1752-0509-5-S3-S10)

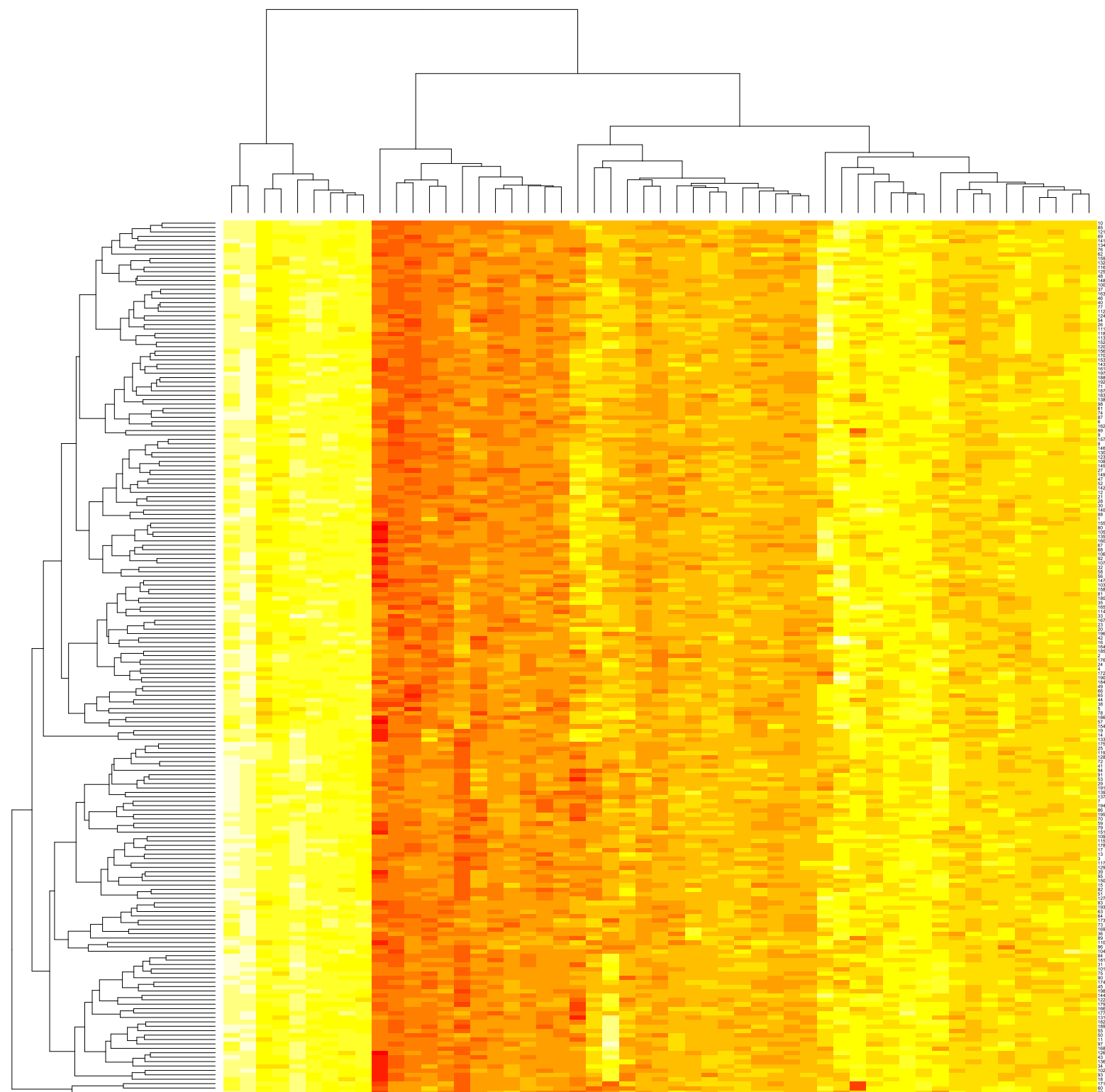

Supplement: Additional file 2 — SC-based gene signature based hierarchical clustering analysis of breast cancer microarray dataset by using SC-based gene signature. According to the gene signature identified by SC, hierarchical clustering analysis is performed on the breast cancer microarray dataset, GSE7390, which include 198 breast cancer patients with various pathologic parameters. [file 1752-0509-5-S3-S10-S2.pdf]
